# Common and Low Frequency Variants in *MERTK* Are Independently Associated with Multiple Sclerosis Susceptibility with Discordant Association Dependent upon HLA-DRB1*15:*01* Status

**DOI:** 10.1371/journal.pgen.1005853

**Published:** 2016-03-18

**Authors:** Michele D. Binder, Andrew D. Fox, Daniel Merlo, Laura J. Johnson, Lauren Giuffrida, Sarah E. Calvert, Rainer Akkermann, Gerry Z. M. Ma, ANZgene, Ashwyn A. Perera, Melissa M. Gresle, Louise Laverick, Grace Foo, Marzena J. Fabis-Pedrini, Timothy Spelman, Margaret A. Jordan, Alan G. Baxter, Simon Foote, Helmut Butzkueven, Trevor J. Kilpatrick, Judith Field

**Affiliations:** 1 Multiple Sclerosis Division, The Florey Institute of Neuroscience and Mental Health, Parkville, Victoria, Australia; 2 Department of Anatomy and Neuroscience, University of Melbourne, Parkville, Victoria, Australia; 3 Bioinformatics Core, The Florey Institute of Neuroscience and Mental Health, Parkville, Victoria, Australia; 4 Department of Medicine, University of Melbourne, Parkville, Victoria, Australia; 5 Western Australian Neuroscience Research Institute, Nedlands, Western Australia, Australia; 6 Comparative Genomics Centre, James Cook University, Townsville, Queensland, Australia; 7 John Curtin School of Medical Research, Australian National University, Acton, Australian Capital Territory, Australia; Georgia Institute of Technology, UNITED STATES

## Abstract

Multiple Sclerosis (MS) is a chronic inflammatory demyelinating disease of the central nervous system. The risk of developing MS is strongly influenced by genetic predisposition, and over 100 loci have been established as associated with susceptibility. However, the biologically relevant variants underlying disease risk have not been defined for the vast majority of these loci, limiting the power of these genetic studies to define new avenues of research for the development of MS therapeutics. It is therefore crucial that candidate MS susceptibility loci are carefully investigated to identify the biological mechanism linking genetic polymorphism at a given gene to the increased chance of developing MS. *MERTK* has been established as an MS susceptibility gene and is part of a family of receptor tyrosine kinases known to be involved in the pathogenesis of demyelinating disease. In this study we have refined the association of *MERTK* with MS risk to independent signals from both common and low frequency variants. One of the associated variants was also found to be linked with increased expression of MERTK in monocytes and higher expression of MERTK was associated with either increased or decreased risk of developing MS, dependent upon HLA-DRB1*15:*01* status. This discordant association potentially extended beyond MS susceptibility to alterations in disease course in established MS. This study provides clear evidence that distinct polymorphisms within *MERTK* are associated with MS susceptibility, one of which has the potential to alter *MERTK* transcription, which in turn can alter both susceptibility and disease course in MS patients.

## Author Summary

Multiple sclerosis (MS) is the most common neurological disease of young Caucasian adults. Oligodendrocytes are the key cell type damaged in MS, a process that is accompanied by loss of the myelin sheath that these cells produce, resulting in demyelination and ultimately in secondary damage to nerve cells. Susceptibility to MS is strongly influenced by genes, and over 100 genes have now been linked with the risk of developing MS. However, surprisingly little is known about the biological mechanism by which any one of these genes increases the probability of developing MS. In this study we have explored in detail the links between one known MS risk gene, *MERTK*, and MS susceptibility. We found that a number of different alterations in the *MERTK* gene are independently associated with the risk of developing MS. One these changes was also linked with changes in the level of expression of MERTK in monocytes, an immune cell type known to be involved in the etiology of MS. In an unexpected result, we found this expression-linked alteration in *MERTK* was either protective or risk-associated, depending on the genotype of the individual at another well known MS risk gene known as *HLA-DRB1*. In addition, we found that not only were alterations in *MERTK* associated with MS susceptibility, but potentially with ongoing disease course, indicating that *MERTK* may be a good target for the development of novel MS therapeutics.

## Introduction

Multiple Sclerosis (MS) is an inflammatory demyelinating disease of the central nervous system (CNS). Although the initiating insult in MS remains unknown, it is clear that the pathology of the disease involves a complex interaction between the immune system, neurons and glia, in which cells of the immune system target oligodendrocytes, ultimately resulting in central demyelination and secondary axonal damage.

A genetic basis for MS susceptibility has long been suggested by the observation of an increased familial risk in twins and in first-degree relatives [1], and there is substantial evidence that the increased risk seen in family members of MS patients is not simply the result of shared environment [1–4]. Although estimates of sibling relative risk (λs) vary, a recent meta-analysis has calculated λs as 16.8, with an overall heritability (h^2^) of 54% [5].

The association of MS susceptibility with specific genes began with studies in the 1970s describing an increase in the frequency of certain human leukocyte antigens (HLA) in MS populations[6–8]. The HLA genes are located on chromosome 6 and include the major histocompatibility class (MHC) I and II loci. Improved methods of subtyping HLA loci, as well as studies with increased sample size, have allowed the identification of an extended HLA haplotype, HLA DRB1*15:*01*, DQA1*0102, DQB1*0602, within the MHC class II region that is strongly associated with the risk of developing MS [9–11]. The association between HLA and MS susceptibility remained for many decades the only convincing association with MS risk. The advent of genome-wide association studies (GWAS) in the last decade has significantly altered the landscape of MS genetics. In 2007, the first MS GWAS detected the first non-HLA loci [Interleukin-2 receptor alpha (*IL2RA*)] to be associated with MS at a genome-wide significance level [12]. A number of GWAS studies of increasing power have since extended the number of loci established as associated with MS risk to 103 [13–15].

One clear finding from MS-GWAS has been that, outside the HLA genes, the majority of the associated variants exert small effects as measured by odds-ratios (ORs <1.10), compared with, for example, the HLA-DRB1*15:*01* (*DR15*) association which has an estimated OR of 3.1 [13]. The combined effect of the known MS-susceptibility loci is estimated to account for only around 28% of the heritability of MS [15]. A number of distinct, and not necessarily exclusive, hypotheses exist as to the nature of the "missing heritability" of MS and other common diseases, including the existence of rare variants of large effect, structural genomic alterations such as copy-number variants (CNVs), or epigenetic modifications, all of which are either poorly captured by GWAS or, in the case of epigenetic changes, not captured at all [16]. It is therefore crucial that candidate MS-susceptibility loci identified by GWAS are carefully investigated to identify the biological mechanism linking genetic polymorphism at a given locus to the increased chance of developing MS.

We and others have demonstrated that signalling via the TAM (TYRO3, AXL and MERTK) family of receptor tyrosine kinases (RTKs) profoundly influences the outcome of demyelination. The TAM receptors were identified as a distinct RTK subfamily in 1991 [17], share a common domain structure [18,19], and are activated by two closely related ligands, GAS6 and PROTEIN S (PROS) [20–22]. The TAM receptors were first linked to demyelination in 2008 when we showed that loss of Gas6 leads to increased disease severity in cuprizone-induced demyelination in mice[23]. More recently, work from our group and others has shown that TAM receptor signalling is involved in both the etiology and the pathogenesis of MS. In 2011, we showed that polymorphisms within the *MERTK* gene are associated with MS susceptibility [24], a finding replicated by a large international GWAS [13], and *MERTK* remains on the current list of established MS-risk loci [15]. The MERTK and AXL receptors, as well as the soluble forms of these receptors, have also been found to be upregulated in MS lesions, and may be correlated with extended lesion activity [25]. Alterations in the circulating levels of the TAM ligands, GAS6 and PROS, have been detected in MS patients, and the level of circulating PROS has been associated with severity measures in MS [26].

These data indicate that *MERTK* is not only an important susceptibility gene for MS, but it could potentially have an ongoing role in determining disease severity. We therefore performed fine-mapping studies to refine the genetic association within this locus in order to identify biologically relevant variants within *MERTK*. We identified both rare and common variants within the *MERTK* gene independently and significantly associated with the risk of developing MS. Two of the associated variants were found to operate in *trans* with the *HLA-DRB1* locus, with one SNP showing discordant association depending upon DR15 status. In exploring the functional basis of the associations, we found that associated variants were also expression quantitative trait loci (eQTLs) for *MERTK* in human monocytes, a cell type central to MS pathology. Finally, we provide evidence that the *MERTK* MS risk-associated variants may also alter disease course in established MS.

## Results

### Identification of a low-frequency SNP within *MERTK* associated with MS susceptibility

We have previously identified SNPs within the *MERTK* gene that are associated with the risk of developing MS [24], and one of the SNPs we identified (rs17174780) was the lead SNP in a large study by the IMSGC [13], confirming the association of *MERTK* with MS susceptibility. As is frequently found for GWAS-identified risk variants, all the risk-associated SNPs within the *MERTK* gene identified in the initial discovery phase were common, none were within exons, nor were they located within any obvious regulatory regions. In an attempt to identify functionally relevant variants underlying the association of *MERTK* with MS susceptibility, we conducted a follow-up fine-mapping study in which SNPs located within and adjacent to a number of known MS susceptibility genes, including *MERTK*, were genotyped to better define the most associated genetic variations leading to increased risk of developing MS [27].

**Fig 1:**
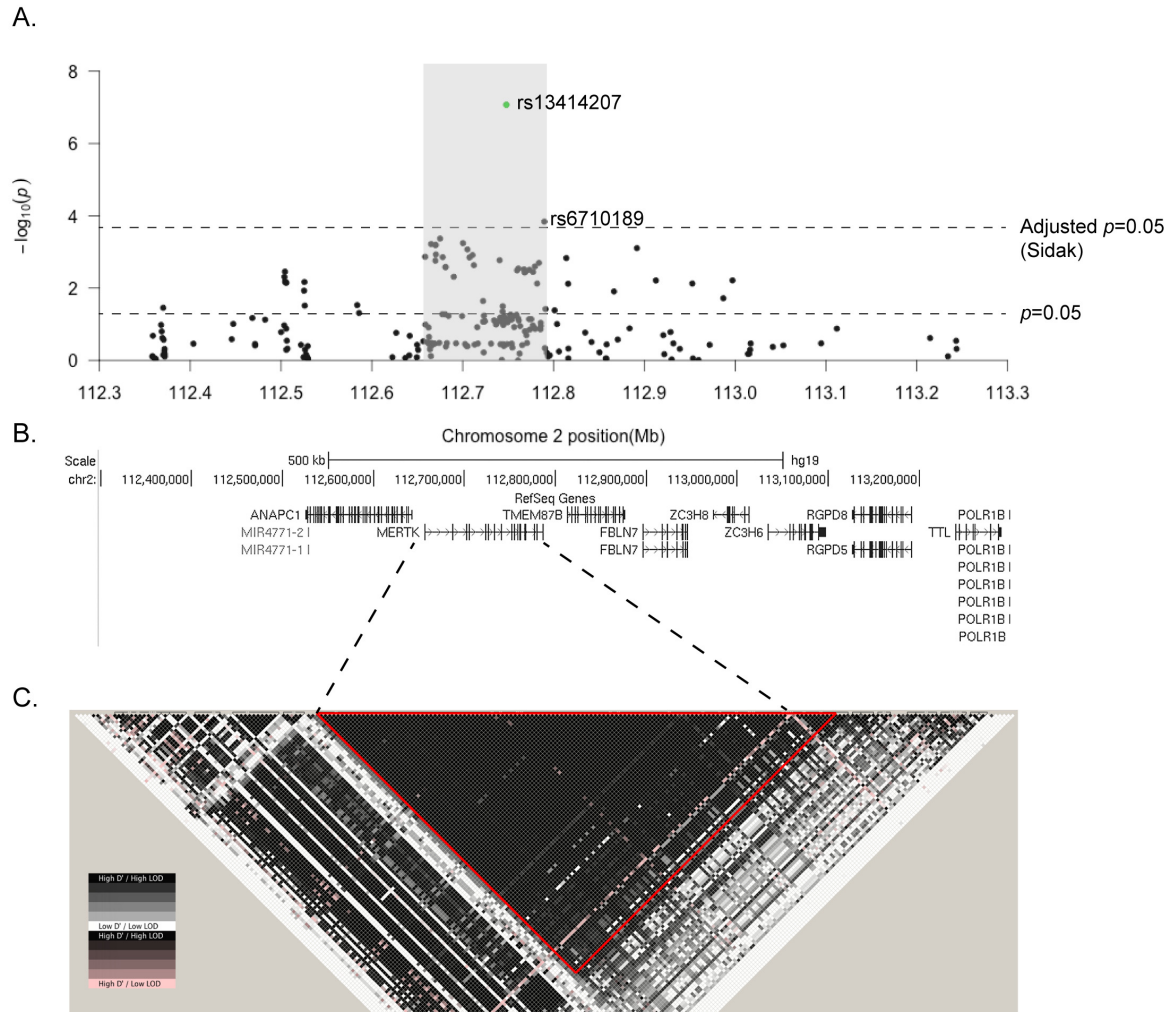
A low-frequency SNP within *MERTK* is significantly associated with MS susceptibility. 124 SNPs on chromosome 2 were directly genotyped in 3268 MS cases and 3579 controls. (A) The negative log of the unadjusted *p*-value of each SNP is plotted against the relative position on chromosome 2, with a schematic of the gene structures shown underneath (B). Two SNPs, both within *MERTK*, reach significance using the Sidak adjustment for multiple testing at a nominal α = 0.05. Association *p*-values were determined using a Chi-square test. (C) Schematic of the pattern of LD across the whole region, with a large single block of high LD (D'>0.99), including the whole of the *MERTK* gene, highlighted with a red triangle.

Included in this follow-up study were 239 SNPs on chromosome 2, including 124 within *MERTK*, selected from dbSNP and the 1000 Genomes project. These SNPs were genotyped in 3268 MS cases and 3579 controls and analysed for association with MS. We identified a peak of association within the *MERTK* region on chromosome 2, with 36 SNPs within the *MERTK* gene showing suggestive association (*p*<0.05), including 2 SNPs significantly associated with MS susceptibility following adjustment for multiple testing (Fig 1A, *p*<2.2 x 10^−4^). The SNPs within the *MERTK* gene that were associated with MS susceptibility (p<0.05), included 8 of the 10 most associated SNPs on chromosome 2 (Table 1). The SNPs within *MERTK* were contained within a region of high linkage disequilibrium (LD) that included the previously associated rs17174870 (Table 1, Fig 1B). The lead SNP within this region, rs13414207, was strongly associated with the risk of developing MS (*p* = 8.5 x 10^−08^), with an OR of 1.6 (Table 1). In contrast to the majority of variants previously associated with MS susceptibility[15], the risk associated allele at the lead SNP is low frequency [minor allele frequency (MAF)<0.05]. We also identified a striking over-representation of individuals homozygous for the risk allele at rs13414207 amongst cases (n = 14) compared with healthy controls (n = 3) amongst the 3268 people with MS and 3579 controls that we assessed, suggesting an allele dosage effect, which fits with a number of potential models (Table 2).

**Table 1:** Top 10 SNPs within *MERTK* associated with Multiple Sclerosis susceptibility.

SNP ID	Position^a^	Minor Allele	Major Allele	MAF^b^ cases	MAF controls	Odds Ratio^c^	Unadjusted *p*-value
rs13414207	112,748,053	A	G	0.04893	0.03082	1.618	8.5x10^-08^
rs6710189	112,789,792	G	A	0.2704	0.242	1.161	0.000145
rs1516639	112,750,085	G	C	0.4753	0.4452	1.165	0.000427
rs884448	112,700,328	A	T	0.4736	0.4442	1.126	0.000572
rs17174870	112,665,201	T	C^d^	0.2219	0.2469	0.87	0.000607
rs1400322	112,670,185	G	A	0.4758	0.4466	1.125	0.000637
rs1400321	112,670,046	A	C	0.2241	0.2491	0.871	0.000649
rs3761700	112,705,185	C	A	0.473	0.4444	1.122	0.000854
rs1400323	112,670,461	C	A	0.4749	0.4471	1.118	0.001179
rs4848901	112,710,828	A	G	0.472	0.4444	1.118	0.001222

a. SNP positions are relative to the Human February 2009 (GRC37/hg19) assembly.

b. MAF = minor allele frequency.

c. The odds ratio applies to the minor allele. d. These alleles are inferred from data obtained from sequencing the opposite strand but are presented as CT to maintain consistency with the remainder of the study.

**Table 2:** Gene model association of rs13414207 with Multiple Sclerosis.

Test	Minor Allele	Major Allele	Affected	Not affected	*p*-value
Genotype	A	G	14/284/2890	3/207/3246	4.84x10^-07^
Trend	A	G	312/6064	213/6699	1.24x10^-07^
Allelic	A	G	312/6064	213/6699	8.50x10^-08^
Dominant	A	G	298/2890	210/3246	5.36x10^-07^
Recessive	A	G	14/3174	3/3453	0.0045

### *MERTK* SNPs define MS risk and protective haplotypes associated with MS susceptibility

**Fig 2:**
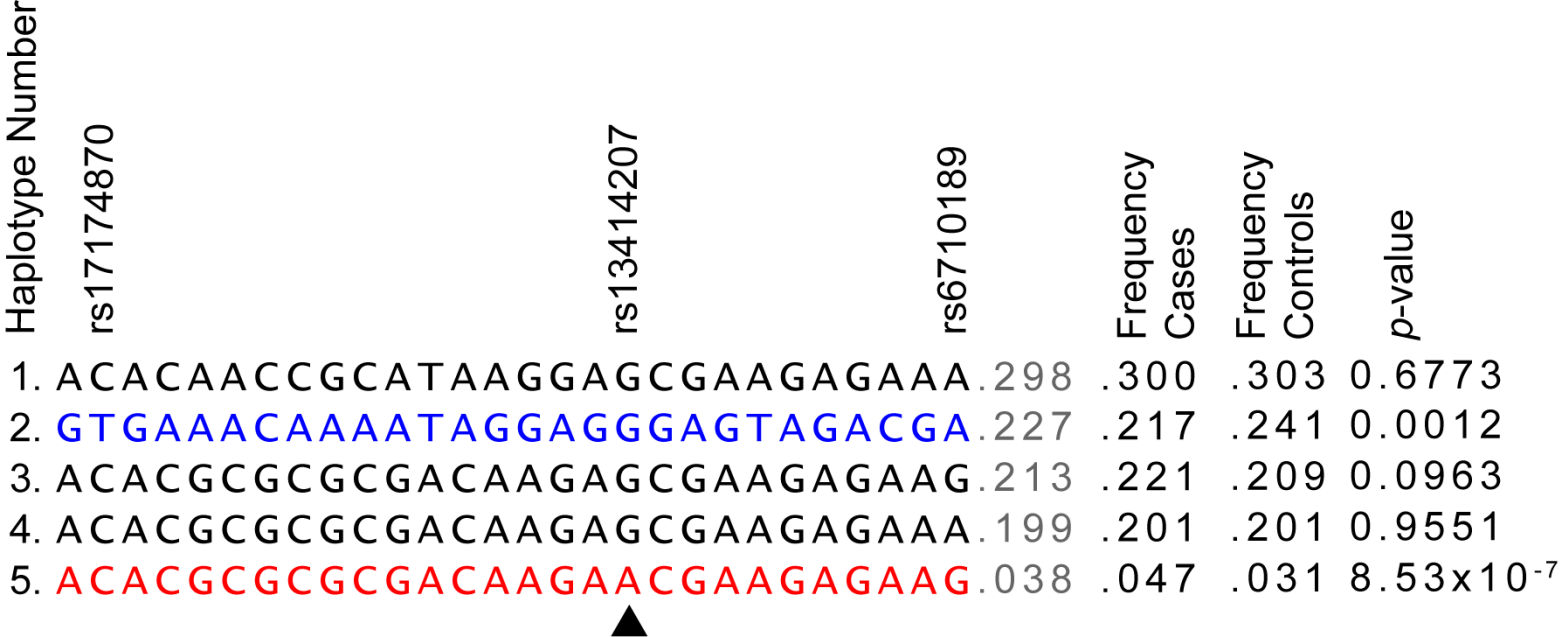
SNPs within *MERTK* define both risk and protective haplotypes associated with MS susceptibility. 28 SNPs within the *MERTK* gene form a single block of very high LD (D'>0.99, LOD≥2). The five most frequent haplotypes (population frequency >1%) are shown in this schematic, along with the *p*-value of association of each haplotype with MS susceptibility as determined using a Chi-square test. Arrowhead indicates the haplotype-tagging allele of rs13414207 in haplotype 5. The alleles presented for rs17174870 are inferred from data obtained from sequencing the opposite strand but are presented as CT to maintain consistency with the remainder of the study.

Given the substantial LD within the region, we examined the relationship of the lead SNP to other SNPs within the *MERTK* gene that showed suggestive association with MS susceptibility. We found that 28 SNPs form a single block of very high LD (D'>0.99; LOD≥2) containing 5 haplotypes with a frequency of >1% (Fig 2). The minor allele of the lead SNP (rs13414207) tags a low frequency haplotype that is significantly associated with the risk of developing MS (Fig 2, *p* = 8.53 x 10^−07^). This risk haplotype also contains variants previously shown to carry MS risk in other studies, including the risk allele of rs17174870 [13,24], as well as a newly identified risk SNP (rs6710189). However, these SNPs do not appear to be independently associated with MS susceptibility outside of this haplotype (Fig 2).

In addition to the haplotype significantly associated with risk, a separate haplotype is significantly more frequent in controls compared with cases, forming an apparent protective haplotype (*p* = 0.0012). As this apparently protective haplotype is tagged by the opposite alleles of the SNPs significantly associated with risk, it may be the effect is not truly protective but represents the absence of risk.

### Haplotype-based variant identification using whole-genome and resequencing strategies

The data accumulated from the fine-mapping strategy suggest that we had either identified a functionally relevant (causal) variant within the *MERTK* gene, or alternatively that we have identified a variant that is in very strong LD with a causal variant. The lack of obvious predicted structural or regulatory alteration resulting from variation at rs13414207 makes the latter more probable. We used the high LD and the strong genotype effect as the basis for two complementary strategies to identify potential causal variants within the *MERTK* gene, with a particular focus on low frequency and novel variants. Specifically, we identified individuals who were homozygous for the risk-associated haplotype (n = 17). We designed and validated a series of overlapping amplicons (n = 31) and employed long-range PCR to amplify the entire *MERTK* gene plus ~10kb upstream and downstream of the gene (Chr2:112,645,298–112,789,127 on GRch37/hg19 build). Amplicons for each individual were pooled at equimolar amounts and sequenced. As it was hypothesised that this strategy would identify a large number of variants, many of which would not be associated with MS susceptibility, we also included a number of individuals homozygous for other identified haplotypes to allow for prioritisation of variants for follow-up association testing (Table 3).

**Table 3:** Individuals used for resequencing of *MERTK*.

Haplotype	rs13414207 genotype	Cases	Healthy controls
2	GG	0	10
3	GG	20	12
4	GG	0	6
5	AA	14	3

As targeted sequencing strategies based on amplification and using paired-end next-generation sequencing technologies are not ideal for detection of large insertions/deletions (in-dels) and other large structural variants, we also undertook whole-genome sequencing (WGS) of a single individual homozygous for the risk haplotype.

**Fig 3:**
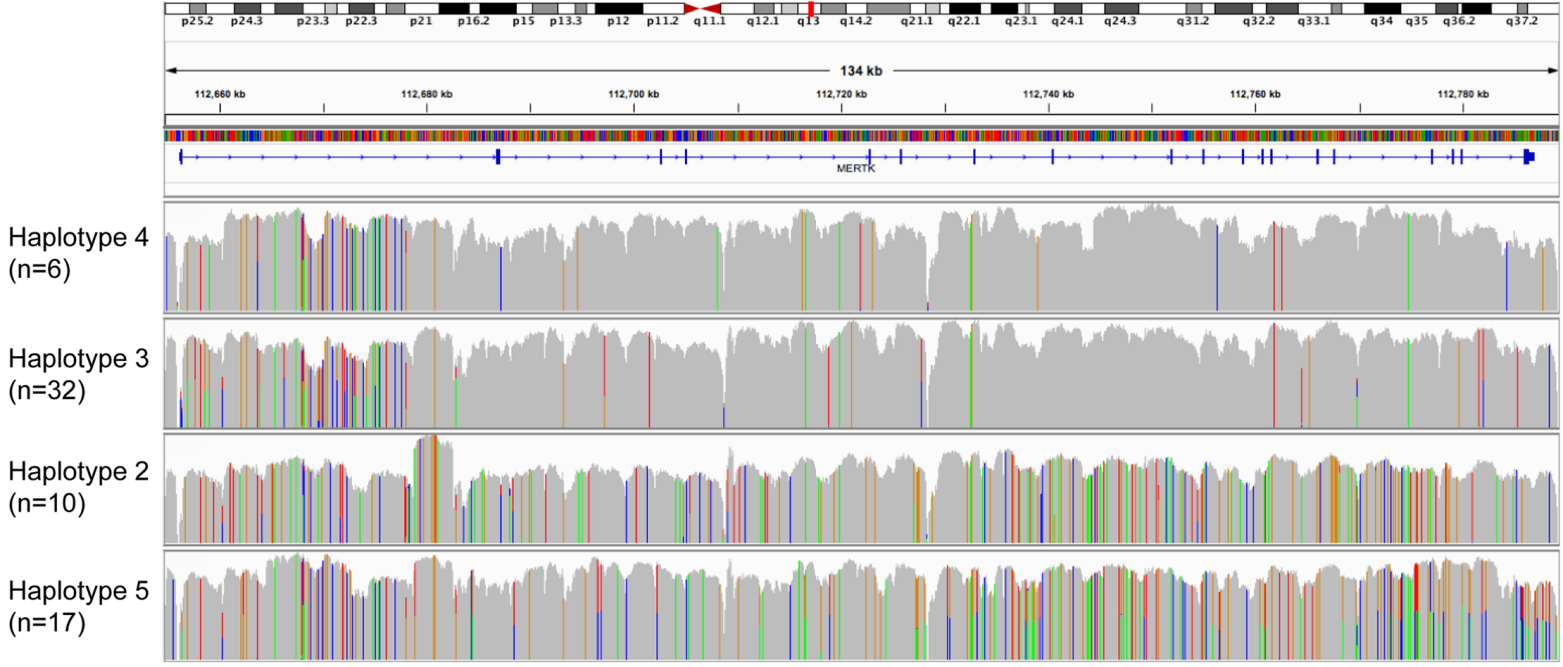
Heat map of variants found in *MERTK* grouped according to haplotype. The sequence of each group following resequencing was compared with the reference genome (GRCh37/hg19). Coloured lines indicate a base that is variant compared with the reference genome. Mapping of the groups shows that haplotype groups 3 and 4 were most closely related to the reference sequence, showing many invariant nucleotides. Conversely haplotype groups 2 and 5 showed the greatest differences to the reference sequence and an apparently close relationship, sharing many variants.

Both strategies produced high quality data, with a sequencing depth of 35-40x (WGS) and 50x for >96% bases and 100x for >90% bases (targeted strategy), and the vast majority of variants (>99%) were identified in both methods. The WGS strategy was successful in identifying two variants that failed to map using the targeted method. The first of these variants was a long repeat expansion within intron 1 (chr2:112,682,854; build hg19) from the reference TA_8_T_21_ to TA_21_T_>26<200_. The second was a retrotransposon insertion polymorphism (RIP) involving an insertion of an AluYf4 retrotransposon in intron 4 (chr2:112,716,560; hg19). Overall we identified 580 variants (SNPs, in-dels and larger variants) within or nearby the *MERTK* gene in individuals homozygous for the risk haplotype (Table 4). As our resequencing strategy was focused upon the risk haplotype, it is not surprising that a comparison of the variation pattern compared with reference sequence showed a significant number of variants within this group (Fig 3). In contrast, the haplotype 3 group, despite sharing many alleles with haplotype 5 in our initial analysis of 28 SNPs (Fig 2) does not overall share many variants with the risk haplotype in the expanded resequencing analysis (Fig 3). Conversely, the haplotype 2 group also contains many non-reference alleles, many of which are shared with the risk haplotype (Fig 3).

**Table 4:** Summary of variants identified in *MERTK*.

Variant Type	Method of Identification	All	Intronic/ Exonic	Novel (exonic)	Private mutations
SNP	WGS^b^/Targeted NGS^c^	337	332/5	88 (0)	44
In-Dels^a^	WGS/Targeted NGS	241	241/0	201 (0)	29
Long Repeat expansion	WGS	1	1/0	1(0)	0
Large In-Del	WGS	1	1/0	1(0)	0
TOTAL		580	575/5	291 (0)	73

a. In-del = Insertion-Deletion.

b. WGS = Whole Genome Sequencing.

c. NGS = Next-Generation Sequencing

We then used a number of methods to prioritise variants for downstream functional analysis and association testing. As we were, *a priori*, attempting to identify polymorphisms in *MERTK* that showed population level association with MS susceptibility, all private mutations were excluded from further analysis. Secondly, any SNPs previously tested in other large data sets, including our fine-mapping data set, and found not to be associated with MS susceptibility, were excluded. We then used our control groups, particularly the haplotype 2 group, to exclude a further set of variants that showed a high degree of sharing between the risk and non-risk haplotypes. At the conclusion of this prioritisation strategy, we developed a final list of 74 variants for further analysis and association testing.

### Low frequency and common variants within *MERTK* are independently associated with MS susceptibility

Following the prioritisation of variants for association testing, we then designed genotyping strategies. A number of SNPs failed at the design or QC stage and were unable to be tested for association. Failed variants included the two novel variants identified via WGS. The TA_n_T_n_ proved to be highly variable in length, with longer expansions correlating with the risk haplotype and the presence of the lead SNP (S2 Table), however the repetitive nature of the expansion was refractory to both sequencing and cloning. Although we showed that the RIP AluYf4 insertion was in perfect LD with the lead SNP (S3 Table), the design of our association testing for newly identified variants (see below) did not allow independent association testing of this variant. At the conclusion of the design process a total of 52 variants (SNPs and in-dels) were taken to association testing.

A total of 1500 cases and an equivalent number of controls were randomly selected from the original fine-mapping data set, excluding the samples used for identification of variants, plus a small number of newly acquired samples. A completely independent sample was not available for association testing, as the original dataset comprised a substantial portion of all available MS cases in Australia and New Zealand. Given these limitations, and the need to specifically exclude all known homozygotes for our original lead SNP (and thus the AluYf4 RIP) it was not possible to independently confirm association of this lead SNP with MS susceptibility, although both rs13414207 and rs17174870 were included in the testing for examination of haplotype structure.

**Fig 4:**
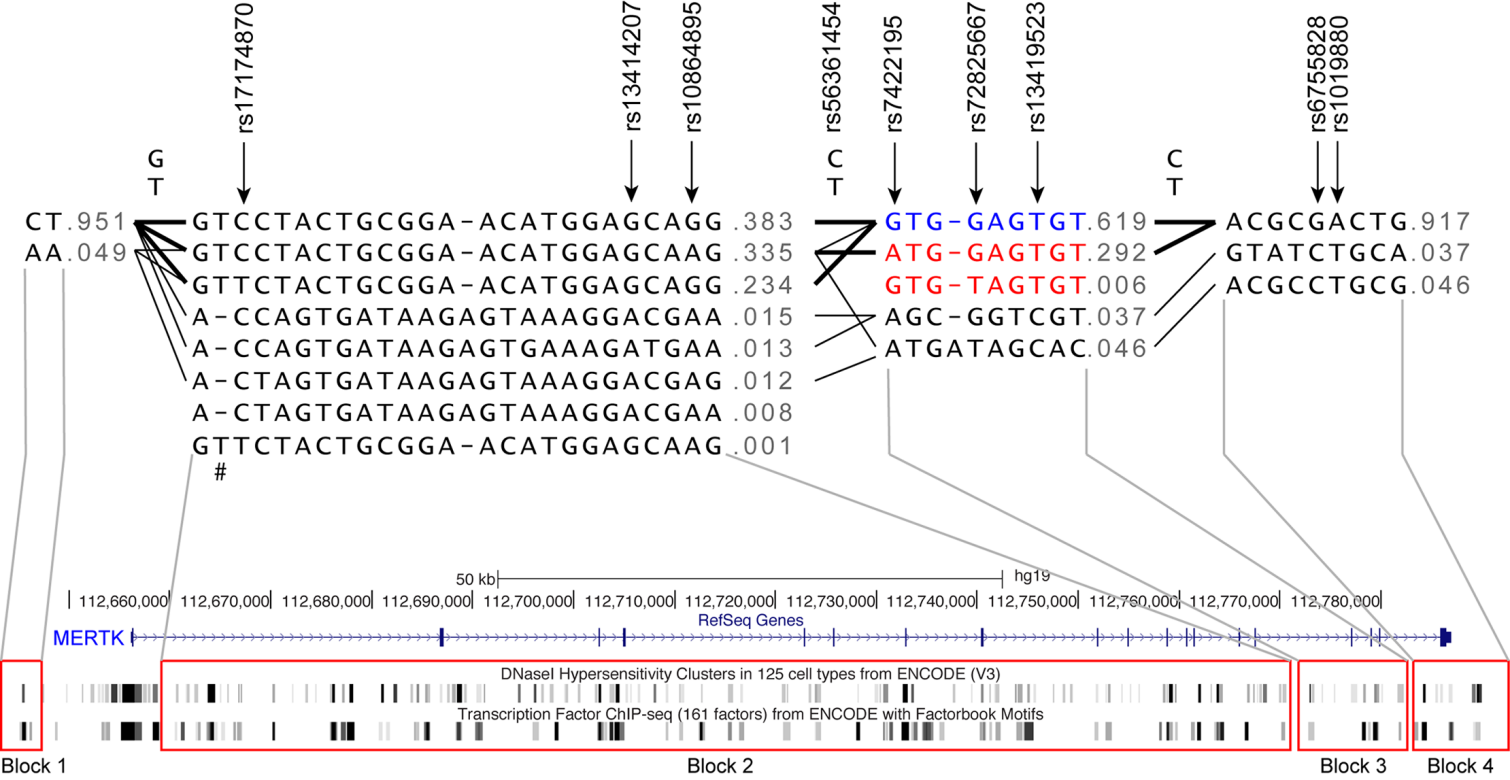
The haplotype structure of variants identified in *MERTK*. The 52 variants in *MERTK* genotyped for association with MS susceptibility fall into 4 separate blocks. Haplotype blocks are connected with thick lines if connections are observed in >10% samples and thin lines if connections are observed in >1% samples. A schematic of the *MERTK* gene is shown underneath indicating the relationship of the blocks to the physical structure of *MERTK*. The haplotypes coloured in red are significantly associated with MS susceptibility (p<0.05) and the haplotype coloured in blue is associated with protection (p<0.05). The *p*-value of association was determined using a Chi-square test. #This variant represents a tri-nucleotide in-del (T/- = TGG; -)

Given that our variant identification strategy was particularly focused on low frequency and novel variants, we first tested association under the genotypic model (2 d.f.). Under this model we observed a number of SNPs, both low frequency and common variants, which showed significant association with MS susceptibility (Table 5). The low frequency SNPs that show association are either contained within the same haplotype block or are connected across blocks (Fig 4), and all have a clear excess of minor allele homozygotes in the affected group (Table 5), suggestive of a recessive effect. Testing these SNPs under a recessive model showed a significant association of rs72825667 with MS susceptibility (*p* = 0.019, OR = 2.3) and this SNP also tags a significantly associated haplotype (Fig 4, *p* = 0.046).

**Table 5:** Results of the association testing for variants identified in *MERTK*.

SNP ID	Position^1^	Minor Allele	Major Allele	MAF	Affected	Unaffected	Chi-square	*p-*value
rs56361454	112774105	T	C	0.34	171/704/615	173/638/683	7.96	0.01868
rs13419523	112781917	C	T	0.083	16/214/1261	7/239/1254	7.041	0.02959
rs7422195	112775064	A	G	0.374	199/717/531	200/661/602	6.974	0.03059
rs6755828	112787215	C	G	0.083	16/216/1257	7/236/1250	6.635	0.03625
rs72825667	112779732	T	G	0.052	25/114/1353	11/124/1361	6.554	0.03775
rs10198880	112787805	T	A	0.083	15/215/1259	7/237/1254	5.995	0.0499

**Fig 5:**
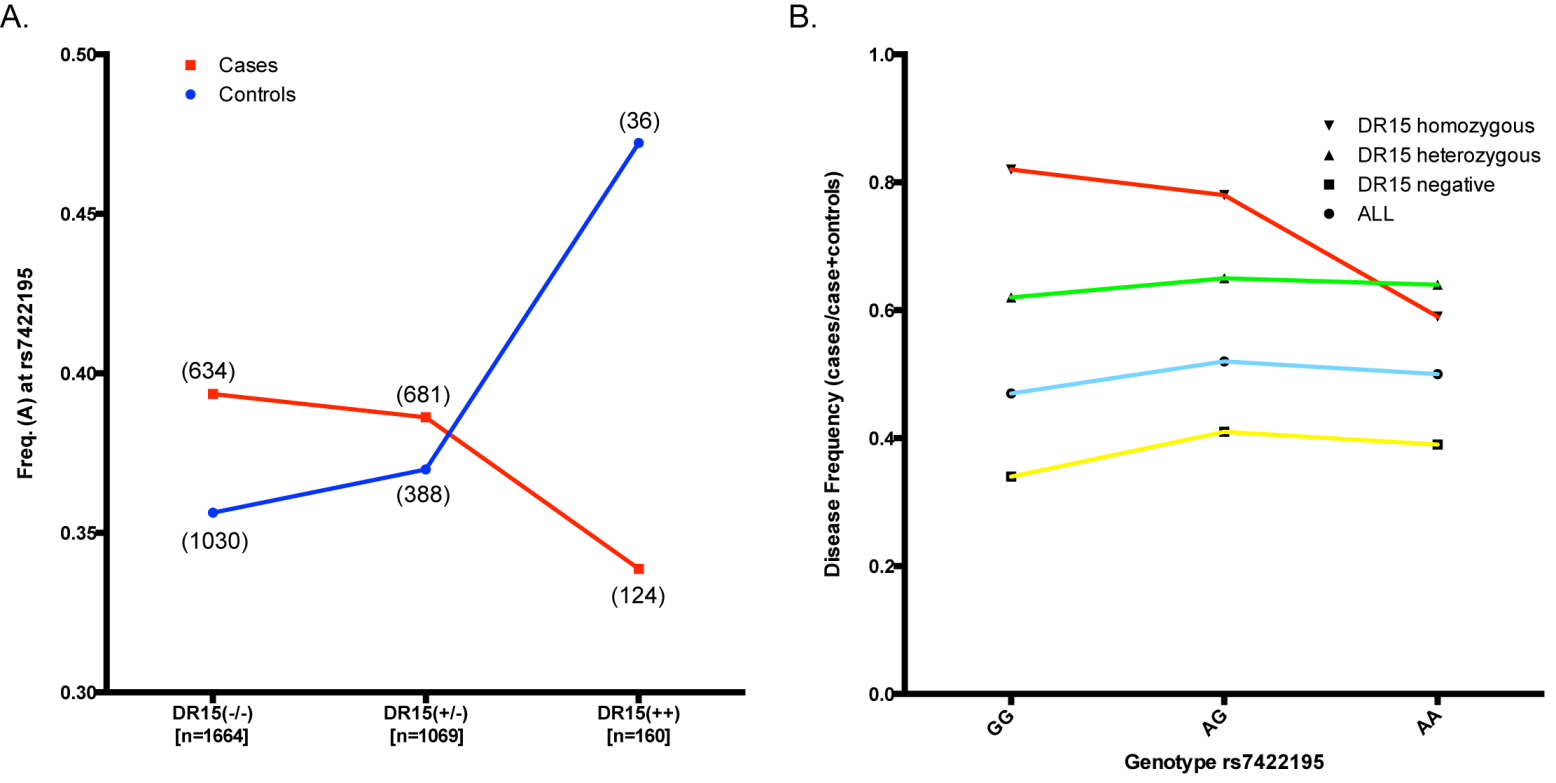
Discordant effect of rs7422195 in the presence or absence of HLA-DRB1*15:*01*. All samples (n = 3000) were first stratified according to the number of *DR15* alleles then by genotype at rs7422195. (A) The frequency of the A-allele of rs7422195 was calculated for cases and controls within each *DR15* genotype group, showing a clear decrease in the frequency of the A-allele with increasing copies of *DR15* within MS cases, and the opposite effect in healthy controls. The total number of samples within each *DR15* genotype group is included below the group name on the x-axis, with the number of individuals used to calculate each point represented in brackets on the graph. (B) Disease frequency for each group was calculated as the number of MS cases divided by the total number of cases and healthy controls for each genotype. The minor allele at rs7422195 shows an increase in the disease risk in the absence of *DR15*, but a clear decrease in the disease frequency amongst individuals carrying two copies of *DR15*.

Given that MERTK is expressed on a number of immune cells which also express MHC II molecules, and which have a well characterised association with MS susceptibility, we wished to determine if any of the *MERTK* variants identified through resequencing showed an interaction with DR15, the predominant risk allele within the HLA locus. We therefore tested all 52 SNPs for interaction with DR15 and in addition to the low frequency variants associated with MS risk, we observed an apparently independent effect of other common SNPs that also showed a statistically significant interaction with *DR15* (Table 6). We therefore separately assessed the association of these SNPs in the *DR15* negative and *DR15* homozygote population and found that whilst these SNPs were both associated in the *DR15* negative population one of these SNPs, rs7422195, was associated in both populations, but the association was discordant, with the opposite allele associated with risk in the *DR15* negative versus DR15 positive group [Table 6, DR15 negative *p* = 0.0383 (A-allele); DR15 homozygous p = 0.0387 (G-allele)]. This SNP also tags a significantly associated haplotype (Fig 4, *p* = 0.049), but importantly tags a different haplotype to that tagged by rs72825667, suggesting an independent effect. When we examined the disease frequency in populations stratified by genotype at rs7422195, it is clear that the minor allele is associated with risk in the *DR15* negative population, but in the smaller *DR15* homozygote population, where the baseline disease rate is quite high, two copies of the minor allele has a clear protective effect (Fig 5).

**Table 6:** Association of *MERTK* SNPs in populations stratified by *DR15* status.

SNP ID	Position^1^	*DR15* Interaction *p*-value	Population	MAF cases	MAF controls	*p*-value	Odds Ratio
rs7422195	112775064	0.0296	*All subjects*	0.3853	0.3626	0.06516	
			*DR15 negative*	0.3935	0.3563	0.0383 (CHISQ)	1.172
						0.00522 (Interaction Model)	1.228
			*DR15 heterozygous*	0.3862	0.3698	0.4539 (CHISQ)	1.072
						0.8547 (Interaction Model)	1.015
			*DR15 homozygous*	0.3387	0.4722	0.03872 (CHISQ)	0.5725
						0.1594 (Interaction Model)	0.7906
rs10864895	112764394	0.0286	*All subjects*	0.3922	0.3743	0.1841	
			*DR15 negative*	0.4	0.3665	0.05 (CHISQ)	1.152
						0.01631 (Interaction Model)	1.187
			*DR15 heterozygous*	0.3956	0.3919	0.8649 (CHISQ)	1.016
						0.5595 (Interaction Model)	1.047
			*DR15 homozygous*	0.3373	0.4583	0.05981 (CHISQ)	0.6015
						0.1082 (Interaction Model)	0.7688

### MS susceptibility variants in *MERTK* are associated with altered expression in human monocytes

**Fig 6:**
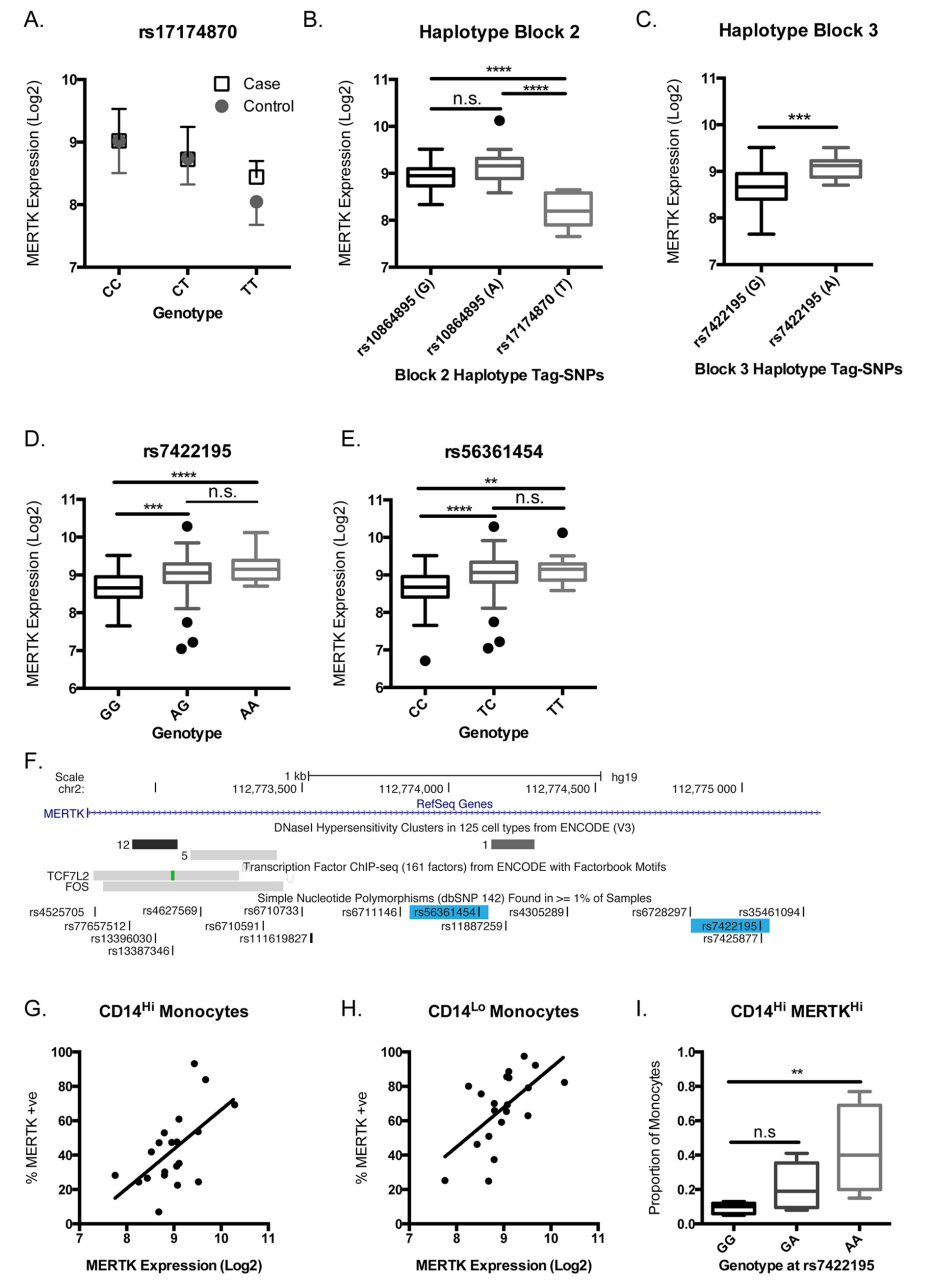
Expression of MERTK in monocytes is genotype dependent. Immune cell subsets were purified from peripheral blood mononuclear cells obtained from MS cases and healthy controls using magnetic cell sorting. (A) Gene expression was measured using the Affymetrix Human ST1.0 array. The genotype for each participant was determined using Immunochip (Illumina) and expression data stratified by genotype at rs17174870. Expression of the *MERTK* gene in monocytes was significantly associated with genotype (*p* = 2.215 x 10^−5^, *p*_*adj*_ = 0.006) but not with phenotype (*p*>0.05). We then stratified individuals carrying various combinations of the haplotype blocks identified in association testing and stratified expression for individuals homozygous for those in block 2 (B) and block 3 (C), with the tag-SNP and relevant allele shown for each haplotype. (B) Individuals homozygous for the haplotype tagged by rs17174870(T) showed significantly lower *MERTK* expression compared with haplotypes A or B (*p*<0.0001). (C) Individuals homozygous for the haplotype tagged by rs7422195(A) showed significantly increased expression of *MERTK* compared with the haplotype tagged by the alternative (G) allele. (D) When samples are stratified by rs7422195 expression of *MERTK* is increased with increased copies of the minor (A) allele [Mean expression±SD: 8.654±0.369 (GG), 9.012±0.5514 (AG) *p*<0.001 vs GG, 9.186±0.3434 (AA) *p*<0.0001 vs GG] (E) When samples are stratified by rs56361454 expression of MERTK is increased with increased copies of the minor (T) allele [Mean expression±SD: 8.655±0.4489 (CC), 9.041±0.5529 (CT) *p*<0.0001 vs CC, 9.139±0.3603 (TT) *p*<0.01 vs CC]. (F) Schematic of the genomic region surrounding rs7422195 and rs56361454 showing transcription factor binding sites and DNase sensitive regions. MERTK expression on the surface of monocytes was determined using flow cytometric analysis of whole blood and correlated with MERTK gene expression. MERTK surface expression was significantly correlated with gene expression in (G) CD14^Hi^ (r^2^ = 0.3434, *p =* 0.0066) and (H) CD14^Lo^ (r^2^ = 0.3624, *p* = 0.005) monocytes. (I) The proportion of CD14^Hi^MERTK^Hi^ monocytes is increased with increasing dose of the minor (A) allele of rs7422195 (*p*<0.01 GG vs AA). All grouped expression data are plotted as Tukey box and whiskers.

The data above clearly link polymorphisms within the *MERTK* gene to MS susceptibility, but due to the multiple independent association signals and high LD within the region, resequencing has not allowed clear determination of a direct disease causing variant. As a complementary approach to dissecting the association of *MERTK* with MS susceptibility, we used an expression quantitative trait loci (eQTL) mapping study to determine the effect of genetic variants upon *MERTK* gene expression in immune cells. We collected peripheral blood mononuclear cells from participants and purified CD14^+ve^ monocytes, CD19^+ve^ B cells, CD4^+ve^ T cells, CD8^+ve^ T cells and CD56^+ve^CD3^-ve^ Natural Killer cells and used Immunochip, which includes the *MERTK* risk-associated SNP rs17174870, to stratify expression based upon *MERTK* genotype. We found that in purified monocytes, but not in the other immune cell types we studied, expression of the *MERTK* gene is strongly correlated with genotype (Table 7, *p* = 2.2 x 10^−5^, FDR *p*_*adj*_ = 0.006). In CD4^+ve^ T-cells there is suggestive association of genotype with *MERTK* expression (Table 7, *p* = 7.6 x 10^−4^, *p*_*adj*_ = 0.091), although as no enrichment for T cells was performed beyond selection for CD4 positivity, the signal in this group could potentially be from CD4^+ve^ monocytes. In contrast, expression was not correlated with phenotype in this population in any cell type (Table 7, *p*>0.05). The risk genotype was associated with higher expression in monocytes, with a clear allele dosage effect (Fig 6A).

**Table 7:** rs17174870 genotype dependent expression of *MERTK* in immune cell subtypes.

Cell type	Number of samples	Unadjusted *p*-value	FDR *q*-value	Genotype-Phenotype Interaction *p*-value
Monocytes	67 cases; 98 controls	2.215 x 10^−5^	0.006	0.4551
B cells	38 cases; 89 controls	0.8052	0.99	0.4756
CD4^+ve^ T cells	43 cases; 88 controls	7.614 x 10^−4^	0.091	0.9277
CD8^+ve^ T cells	56 cases; 74 controls	0.06279	0.69	0.5467
Natural Killer cells	56 cases; 93 controls	0.0385	0.69	0.7777

In order to better define potential functional variants within the *MERTK* gene we then combined our expression data with genotype information obtained from our resequencing strategy. Given that each haplotype block contained multiple potential regulatory regions (Fig 4) we first stratified our samples by haplotype blocks to determine which region was most associated with altered *MERTK* expression. We then compared expression in individuals homozygous for the most frequent haplotypes in blocks 2 (from intron 1 to intron 15) and 3 (from intron 15 to intron 18) (Fig 4). We were unable to compare other blocks or less frequent haplotypes due to insufficient sample numbers. The tag-SNPs within each block, irrespective of their marginal association with MS-risk, were identified and used to stratify haplotypes. When stratified by block 2, we observed significantly lower expression associated with the haplotype tagged by the minor allele (T) at rs17174870 (Fig 6B, *p*<0.0001), but no differences in expression between the two haplotypes tagged by the alleles at rs1084895 (Fig 6B, *p*>0.05). This data suggests firstly that rs1084895 is not likely to be the causal factor for expression differences; and secondly, that the non-risk associated allele at rs17174870 is associated with lower expression, either as a tag-SNP or potentially as the biologically relevant variant.

Conversely, in block 3 (Fig 4), the major haplotype tagged by the minor allele at rs7422195 is associated with significantly higher expression of the *MERTK* gene (Fig 6C, *p*<0.001), implicating this SNP as the functionally relevant variant associated with increased expression of *MERTK*. When all samples were stratified by genotype at rs7422195, we observed the minor allele (A) was significantly associated with increased *MERTK* gene expression (Fig 6D, *p*<0.0001). However, rs7422195 is in very strong LD (r^2^ = 0.99) with rs56361454, a SNP not contained within any block, and when samples are stratified by this SNP, the minor allele (T) is similarly associated with significantly increased expression of *MERTK* (Fig 6E). These data therefore strongly implicate either rs7422195 or rs56361454 as the functionally relevant variant altering *MERTK* expression within monocytes. An examination of the genomic region containing these variants shows that both are within the same 2kb region that contains known transcription factor binding sites for TCF7L2 and FOS, as well as other genomic features associated with transcriptional activity such as DNase hypersensitivity sites, (Fig 6F). However, we cannot formally exclude that rs7422195 may be acting as a tag-SNP for another variant directly affecting *MERTK* transcription, including the TA_n_T_n_ novel repeat expansion identified in this study and for which we could not design a genotyping assay, as the A-allele at rs7422195 appeared to be associated with the presence of larger expansions (S1 Fig).

The data above clearly show that variants within *MERTK* are significantly associated with changes in *MERTK* gene expression. We used flow cytometric analysis to explore whether changes in transcription of the *MERTK* gene were reflected in alterations in the expression of the MERTK receptor on the surface of monocytes. We found that the expression of *MERTK* gene was significantly correlated with the percentage of MERTK-positive cells in both the classical (CD14^Hi^) monocyte population (Fig 6G, r^2^ = 0.34, *p* = 0.0066), and the non-classical (CD14^Lo^) monocyte population (Fig 6H, r^2^ = 0.36, *p* = 0.005). When we stratified the classical (CD14^Hi^) monocyte population by genotype at rs7422195 and determined the proportion of monocytes that were MERTK^Hi^, we found that the minor allele was significantly associated with a 4.5 fold increase in the proportion of CD14^Hi^MERTK^Hi^ monocytes (Fig 6I, *p*<0.001), consistent with the increased transcription of *MERTK* associated with this allele.

### The *MERTK* gene contains a novel alternative final exon, but exon usage is not altered by MS susceptibility variants in *MERTK*

As the initially identified low frequency risk associated SNP (rs13414207) was in perfect LD with an AluYf4 insertion, and such insertions have been shown in other diseases to be associated with exon-skipping [28,29], we wished to test whether risk-associated SNPs within *MERTK* were associated with differential exon usage in monocytes. We therefore purified monocytes from MS cases (n = 5) all of whom were homozygous for rs13414207(A), and which were also homozygous for the AluYf4 RIP. In addition, due to haplotype structure in the region, all patients were also homozygous for the rs7422195(A) allele associated with increased *MERTK* gene expression but homozygous for the low expression associated C-allele at rs56361454. We also purified monocytes from healthy controls (n = 3) all of whom were homozygous for the opposite alleles for the aforementioned SNPs [ie. rs13414207(G)-rs7422195(G)-rs56361454(T)] and who were negative for the AluYf4 RIP. We then extracted mRNA from the purified monocytes and performed RNA sequencing (RNAseq). Whole transcriptome analysis for differential gene expression revealed that *MERTK* transcripts were increased 3.63 fold (*p* = 3.7 x 10^−5^, FDR *p*_*adj*_ = 0.03) in the group homozygous for the rs7422195 allele previously associated with high expression, confirming the association of this allele with increased *MERTK* expression. Conversely, as this group were all homozygous for the (T) allele at rs56361454, which we were previously unable to separate from rs7422195 due to high LD, this data suggests that the minor (A) allele at rs7422195 is the biologically relevant variant linked with *MERTK* gene expression.

**Fig 7:**
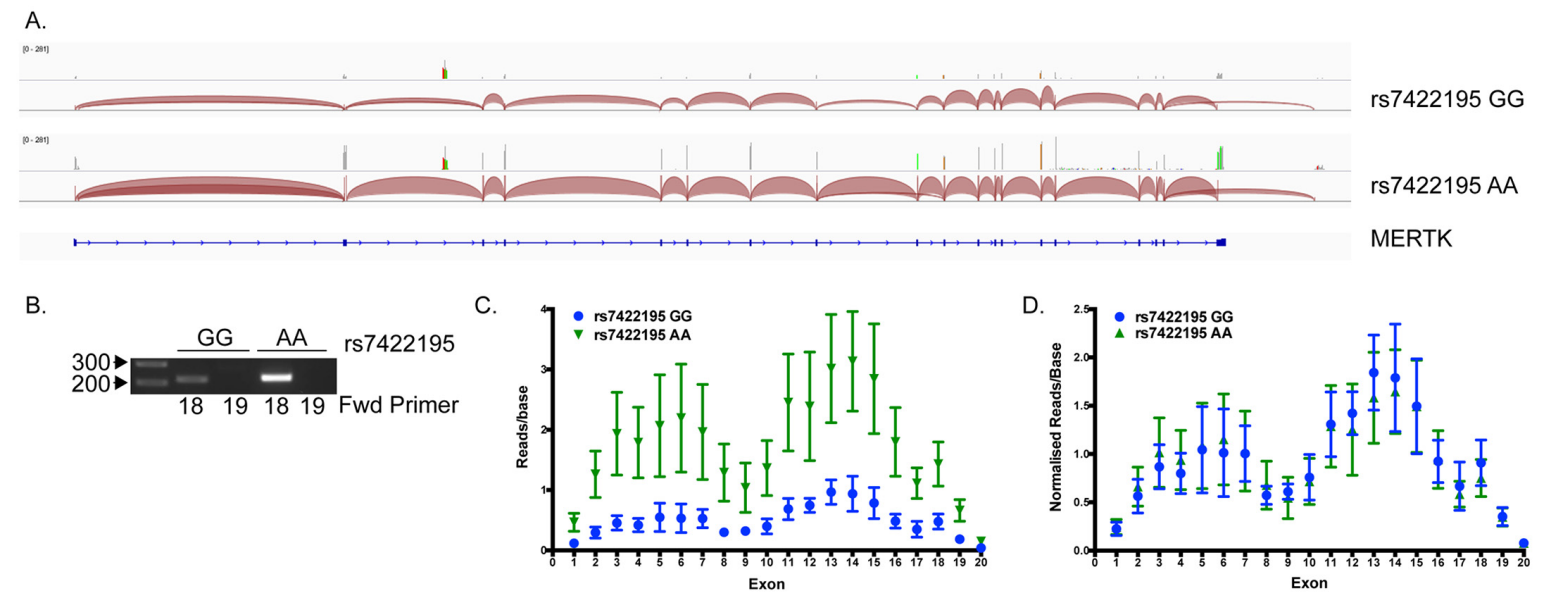
The *MERTK* gene contains a novel alternative final exon but usage is not genotype-dependent. (A) Exon junctions in RNA sequencing data were visualised using IGV_2.3.35. Representative junction maps for a low (rs7422195 GG) and high (rs7422195 AA) expressing sample are shown. Each shows the presence of a junction from exon 18 to a putative novel alternative final exon excluding exon 19. (B) RT-PCR analysis of a high and low expressing sample show the presence of this exon only in combination with exon 18 and not exon 19. (C) Exon usage analysis shows overall increased expression of each exon in samples homozygous for the rs7422195 minor (A) allele, but no difference in exon usage following normalisation for overall expression level (D).

In addition to assessing overall expression effects, we also analysed the transcriptome for altered RNA splicing. We observed no differences in the location of splice junctions in *MERTK* in either the high or low *MERTK* expressing group and the majority of junctions aligned with canonical 19 exons within the *MERTK* gene (Fig 7A). The only exception to this was that all samples contained a junction from the 3' end of exon 18 to a small putative alternative final exon that excluded the canonical final exon 19 (Fig 7A). Alignment of the transcript reads showed the putative final exon to be approximately 1273bp at position 112,796,928–112,798,200 (genome build GRCh37/hg19). We confirmed the inclusion of this alternative exon in some *MERTK* transcripts using reverse transcription PCR (Fig 7B). The inclusion of this exon, and the exclusion of the canonical final exon, would lead to an early stop signal within the tyrosine kinase domain of MERTK and potentially the production of a membrane-bound non-signalling MERTK receptor. However, the inclusion or exclusion of the alternative final exon does not appear to be altered by polymorphisms within the *MERTK* gene, as we observed no significant differences in exon usage between the two groups. Although overall expression of each exon was higher in the high expressing group (Fig 7C), once expression was normalised to account for the 3.63 fold overall expression increase in this group, the pattern of exon usage was the same in the two groups, independent of genotype (Fig 7D).

### *MERTK* risk variants may alter disease course in established MS

**Fig 8:**
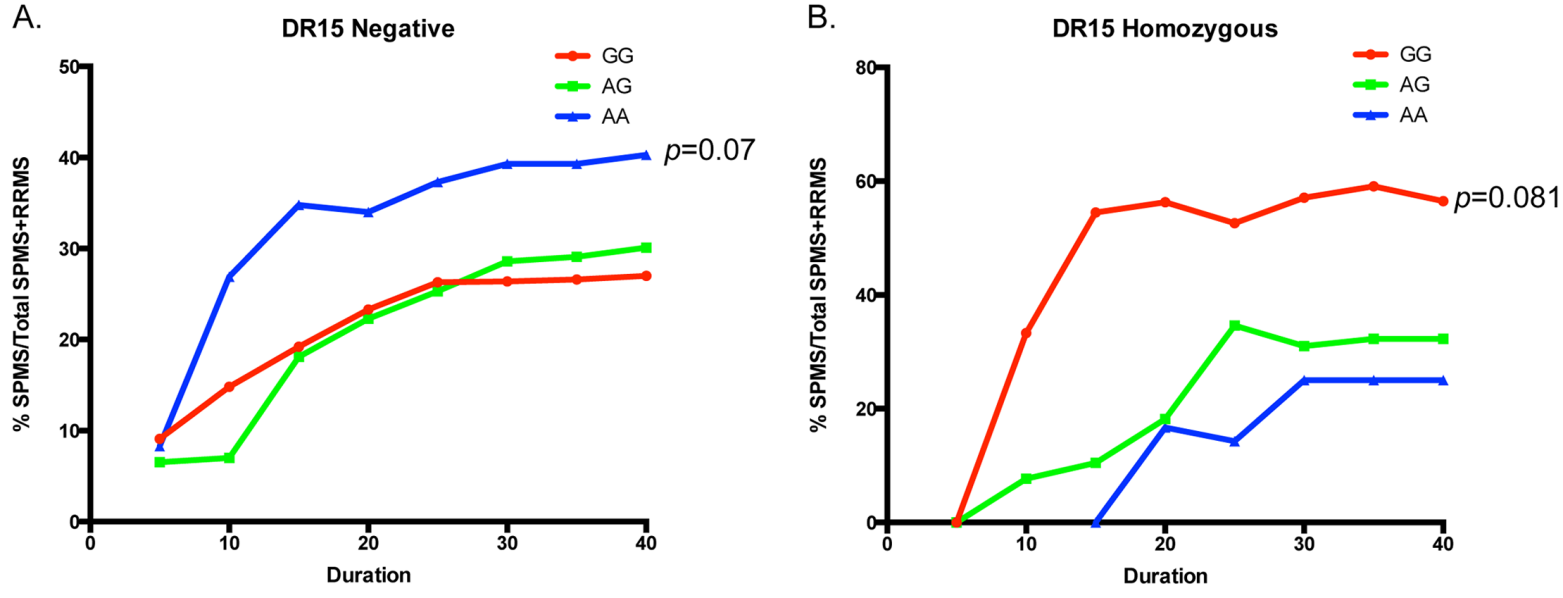
Disease course is altered in the presence of *MERTK* susceptibility-associated variants. Individuals initially presenting with a relapsing-remitting course of MS were stratified by both *DR15* status and genotype at rs7422195. (A) In the presence of the minor (A) allele of rs7422195, *DR15* negative individuals (n = 370) showed a strong trend towards increased probability of progression (*p* = 0.07) (B) In the presence of the major (G) allele of rs7422195 *DR15* homozygous individuals (n = 68) showed a strong trend towards increased probability of progression (*p =* 0.081)

We have previously shown that differential expression of the TAM ligand PROS is associated with altered severity in established MS [26]. We therefore wished to investigate whether the genotype dependent expression changes have an ongoing role in modulating severity in established MS. The onset of progression following a relapsing course of MS is one of the key determinants of clinical outcome [30]. We assessed a subset of our samples (n = 874), all recruited in the same centre (Victorian MS cohort), for the cumulative probability of MS progression over time. We stratified our data by genotype at rs7422195 and divided the cohort into *DR15* negative and *DR15* homozygous groups. We found that in both groups, there was a strong trend towards altered probability of progression dependent upon genotype at rs7422195 (Fig 8) but that, consistent with the association data, the opposite allele was associated with altered progression, such that in the *DR15* negative group, patients homozygous for the risk-associated (A) allele have an increased probability of progression, although the effect does not reach statistical significance (Fig 8A, *p* = 0.07, OR = 1.9, 95% CI 0.948 to 3.87). Conversely, in the *DR15* homozygous group, where the (G) allele is associated with risk, patients homozygous for this allele have an increased probability of progression (Fig 8B, *p =* 0.08, OR = 5.6, 95% CI 0.811 to 38.2).

## Discussion

In this study we have refined the association of *MERTK* with MS susceptibility to independent signals from both common and low frequency variants. We have shown that one of the associated variants, which is an eQTL for MERTK expression, is in *trans* with the *HLA-DRB1* locus, and shows discordant association dependent upon DR15 status. In addition, we have identified a number of low-frequency variants, all contained within the same haplotype, which are associated with MS susceptibility and operate in a recessive manner. Finally, we have shown that polymorphisms within *MERTK* affect not only MS susceptibility, but may also affect severity in established MS.

The main focus of this study was to refine the association of *MERTK* with MS susceptibility and to identify potential causal variants. In the process of this refinement, we identified both common and low frequency variants within *MERTK* independently associated with the risk of developing MS. The variants identified in this study are all in LD with the common variant within *MERTK*, rs17174870, previously identified by us, and replicated by the IMSGC, as associated with MS susceptibility [13,24]. The majority of variants captured in GWAS studies are common (MAF >0.05), and those found to be associated with disease are often intronic and outside any obvious regulatory regions, leading to the speculation that the signals detected in GWAS are in fact the result of synthetic associations between rare variants and common variants [31]. However, a recent study has cast doubt upon this hypothesis, and found limited support for the influence of rare coding sequence variants upon disease susceptibility in a combined analysis of six common autoimmune diseases, including MS [32]. In contrast, it has recently been shown that a rare null mutation in the P2X7 receptor is strongly associated with protection against MS [33]. This study of *MERTK* suggests that, at least in some cases, associations from low frequency variants may underlie the signal observed in GWAS analysis of common variants, and that careful selection of individuals for resequencing is crucial for the detection of the true underlying signal, as has previously been suggested [34].

The lead SNP in our fine-mapping studies, rs13414207, was a low-frequency variant located between exons 7 and 8 within the *MERTK* gene, and was not within any clear regulatory region such as a transcription factor binding site. Resequencing of the *MERTK* gene has successfully identified a number of variants, all in LD with the lead SNP and showing a similar recessive mode of effect. However, we were unable from this study to determine which of the variants identified from the resequencing strategy was the biologically relevant variant. The failure to do so was partially due to the strong LD within the region, and partially due to technical limitations in our ability to genotype individuals for many of the novel SNPs and larger variations such as the AluYf4 RIP and the TA_n_T_n_ expansion. However, although we did not identify a clear causal variant in this region, homozygosity for the risk-associated haplotype containing these low frequency variants shows a substantial increase in the risk of developing MS, with double the risk of MS amongst homozygous individuals. This increased risk appears to be independent of the expression of MERTK, with these recessive-effect SNPs found on haplotypes linked with both low and high MERTK expression. Further work will be required to determine the biological effect of these variants and whether they act independently or if MS risk is associated with overall burden of these low frequency alleles. The two larger variants in particular warrant further investigation, as these types of variants have been linked with other human diseases. For example, a pathogenic retrotransposon insertion in the 3'UTR of the Fukutin gene leads to the development of Fukuyama-type congenital muscular dystrophy [29], whereas an AT_n_ dinucleotide repeat polymorphism within the *CTLA-4* gene has been shown to be protective against development of Graves' Disease in childhood [35].

In addition to the recessive low frequency variants, we also identified other more common variants associated with MS susceptibility, one of which showed discordant association depending upon the *DR15* status of the individual. Discordant associations have been previously reported across different autoimmune diseases (reviewed in [36]), but to our knowledge this is the first such association described within a single disease. In this case, the minor allele at rs7422195 is associated with MS susceptibility in the absence of *DR15*, but this is converted to a protective effect on a *DR15* homozygous background.

What might mediate this discordant effect of rs7422195? We have shown that in monocytes, but not other immune cell types, this SNP is associated with altered expression of MERTK, at both the gene and protein level, and that the minor allele of rs7422195 is associated with increased expression of MERTK. Monocytes are the precursors of a number of cell types, including macrophages and dendritic cells, that have been shown to be central to the etiology of MS (reviewed in [37,38]). We further found that there was suggestive genotype dependent expression of *MERTK* in CD4^+ve^ cells, the majority of which are T cells. Although this association would need to be validated in order to exclude the possibility that the detected altered expression was not the result of contaminating CD4^+ve^ monocytes, it has recently been shown that Mertk is expressed in the Th17^+ve^ subset of CD4^+ve^ T cells in mice following induction of experimental autoimmune encephalomyelitis, a mouse model of MS [39].

MERTK is an important regulator of immune activation, and the expression of MERTK is essential to the maintenance of immune homeostasis—maintaining the balance of immune activation when required and immune suppression following challenge. In animal models it has been shown that the relationship of MERTK to autoimmune disease is complex, and in some cases dependent upon genetic background. For example in the NOD (non-obese diabetic) mouse model of spontaneous diabetes, knockdown of *MERTK* results in resistance to the development of diabetes, and this effect appears to be mediated by bone-marrow derived dendritic cells [40]. In contrast in a different NOD genetic background, in this case in the presence of a transgenic T-cell receptor, deficiency of Mertk leads to an exacerbation of the rate of spontaneous disease [41]. In humans, MERTK expressed on dendritic cells has been shown to be an important negative regulator of T cell activation, whereby MERTK expressed by tolerogenic dendritic cells suppresses T cell activation and proliferation [42]. The ability of MERTK to suppress activated T cells may extend to previously activated memory T cells [42], providing a clear and plausible link between increased MERTK expression and protection against MS susceptibility, as observed in our *DR15* homozygous population: a population in which increased expression of the antigen presenting molecules encoded by the *DR15* haplotype is hypothesised to lead to more efficient presentation of encephalitogenic peptides and increased activation of autoreactive T cells [43]. What is not clear is why this link breaks down in the DR15 negative population, where increased MERTK expression is associated with MS risk. In this negative population, where antigen presentation and subsequent T cell activation may not be such a clear driving force for susceptibility, it is possible that other factors may be at work, perhaps involving similar biological processes to that observed in NOD mice, where deficiency of Mertk reduces susceptibility to spontaneous diabetes.

One of the overarching objectives of studies attempting to define disease susceptibility variants is to identify genes and pathways for intervention. In the absence of good predictors of disease susceptibility, the pathways most amenable to intervention are those still active in controlling clinical course in established disease. In this study we have presented evidence that, in addition to disease susceptibility, MERTK expression associated variants may also be related to severity measures, specifically the rate of conversion from the relapsing-remitting phase of RRMS to progression. We found that, consistent with the discordant association in the presence or absence of *DR15*, the allele associated with high expression of MERTK was associated with a decreased prevalence of MS in *DR15* positive homozygotes, and a higher prevalence of MS in the absence of *DR15*. These data highlight the potential of MERTK as a therapeutic target, although the *DR15* status of the patient would likely determine whether activation or repression of MERTK signalling would be the appropriate intervention.

## Materials and Methods

### Ethics statement

Recruitment of subjects and collection of tissue and DNA was approved by the Melbourne Health Human Research Ethics Committee (Project number: 2013.111), the Eastern Health Human Research and Ethics committee (Reference number: SERP27/1314) and the Australian Bone Marrow Donor Registry Ethics Committee (Project number: 2006/02). All Human Research Ethics Committees which provided approval for this research are guided by national standards as outlined in the National Statement on Ethical Conduct in Human Research (https://www.nhmrc.gov.au/guidelines-publications/e72) issued by the National Health and Medical Research Council (Australia). All cases and controls provided written consent for the use and storage of DNA and tissue samples.

### Study subjects and DNA samples

The 3268 MS cases and 3579 healthy controls genotyped for the fine-mapping component of this study formed part of a larger ANZgene MS loci fine-mapping study, and were phenotyped according to established criteria [27]. To assess for potential population stratification, principal component analysis was performed and genomic inflation was determined to be 1.10, and outlier samples removed as previously described [14]. We estimated the power of the sample used for fine mapping using the online genetic power calculator (http://pngu.mgh.harvard.edu/~purcell/gpc/). The sample was estimated to have high power (>90%) for both variants of moderate frequency with moderate risk (eg. MAF = 0.3, marker = 0.3, D' = 0.99, disease prevalence = 0.001 and relative risk = 1.2; alpha = 0.05), as well as for variants of low frequency with a stronger risk (MAF = 0.05, marker = 0.05, D' = 0.99, disease prevalence = 0.001 and relative risk = 1.6; alpha = 0.05).

A subset of these samples (1500 cases and 1500 controls) were used for association testing of the variants identified in the resequencing component of the study. The subjects used for the resequencing component of the study were excluded from association analysis of identified variants. We used the online genetic power calculator (http://pngu.mgh.harvard.edu/~purcell/gpc/) with the following assumptions and calculated a minimum sample size for 80% power (at alpha = 0.05) of 1465 cases: MAF 0.02 (and "marker" at the same frequency since we predicted that re-sequencing will identify biologically relevant variants that we directly test); D' = 1, disease prevalence = 0.001 and relative risk = 1.618. A small subset of samples used in resequencing were re-recruited for RNA sequencing analysis (n = 5 cases; n = 3 controls).

Subjects (n = 67 MS cases and 98 healthy controls) were recruited for the analysis of expression of MS risk-associated variants as part of a larger study and according to the criteria described previously [44]. These samples were also genotyped as part of the associating testing following variant identification.

### Whole genome sequencing

A sequence-ready DNA library of short sequences for whole genome sequencing was prepared using the TruSeq PCR-free library preparation kit (Illumina, San Diego, CA). The sample library was uniquely barcoded and sequenced on a single lane of an Illumina HiSeq 2000 sequencer (Illumina, San Diego CA).

### Amplicon design and targeted resequencing

PCR amplicons were designed to tile completely the genomic region of the MERTK gene (*GRCh37/hg19* region chr2:112,645,298−112,789,127). Illumina short read sequencing libraries were constructed for each individual DNA sample after targeted capture and amplification of the *MERTK* locus based on the amplicon tiling design. Libraries for sequencing were prepared by the Australian Genome Research Facility (AGRF) and uniquely barcoded. All libraries were then sequenced in a multiplexed fashion within a single lane of an Illumina MiSeq sequencer by AGRF.

### Variant discovery

Sequencing output was demultiplexed and base calling quality scores were verified using FastQC software which confirmed that reads were of uniformly high quality (Q>30). We mapped all reads to the human reference genome build GRCh37/hg19 using the BWA aligner [45]. Across all samples a minimum of 90% of sequence reads mapped successfully to the reference genome, and this resulted in an average read depth of at least 40X in the target (*MERTK*) locus, giving more than sufficient depth to make robust sequence variant calls. We used Picard (http://picard.sourceforge.net) to remove PCR and optical duplicate reads. To call sequence variants [SNPs and insertions-deletions (in-dels)] we used GATK v3.1 [46] and implemented the GATK DNA-sequence variant calling best practice guidelines [47,48].

### Genotyping

Genotyping of the full sample set for the fine-mapping phase was performed using 1536 custom GoldenGate assay according to manufacturer's instructions [27]. Target specific primers for KASP (Kompetitive Allele Specific PCR) genotyping were designed for short variants (SNPs and in-dels) identified during the variant discovery phase and chosen for association testing, with each assay validated using samples of known genotypes. Assays that failed at either the design phase or during validation were then redesigned for the Sequenom MassArray system using iPLEX Gold chemistry, and assays validated using samples of known genotypes. Assays that also failed redesign were discarded. KASP assays were conducted by LGC genomics (Teddington, UK) and Sequenom MassArray genotyping was performed by the Garvan Molecular Genetics Service (Darlinghurst, Australia). All assays were plotted using cluster analysis software [KRAKEN (LGC Genomics, Teddington, UK)] and scored visually to ensure genotypes were correctly assigned. Two large variants not suitable for KASP genotyping (AluYf4 retrotransposon insertion and TA_n_T_n_ repeat expansion) were assessed using PCR in a subset of samples and not included in association analysis. Sequences of primers were as follows: AluYf4 retrotransposon insertion forward (ATCACTGGGCCTGAAATCTG), reverse (CATGCCTTGGCATCACTTTT); TA_n_T_n_ repeat expansion forward (GGGTCCTAGCACCTAACCTG), reverse (CCACGAAACCTACCCTGAAA).

### Association analysis

Prior to testing for association, all markers were assessed for Hardy-Weinberg equilibrium (HWE). Any variants that deviated from HWE (*p*<0.001; S1 Table) in the control or case group were then carefully re-assessed for quality of genotyping by cluster plot analysis and comparison of called genotypes to known genotypes from sequencing data, or to HLA typing where available in the case of HLA-DRB1*15:*01*. Markers that showed evidence of poor quality genotyping were excluded from further analysis.

Association of variants (SNPs and in-dels) was measured using the allelic association test [1 degree of freedom (d.f)] or the genotyping test (2 d.f.) as appropriate using PLINK (v1.07; http://pngu.mgh.harvard.edu/~purcell/plink/).

We used standard logistic regression (R v3.1.2; https://www.r-project.org/) to identify potential interactions with *DR15* in the context of association. To test for statistical interaction between rs7422195 genotype and *HLA-DR15* status in the context of MS disease association, we constructed a standard logistic regression model with a response (output) term for MS disease status, and covariate (input) terms for rs7422195 genotype, *HLA-DR15* status and the corresponding multiplicative interaction term between these two covariates (the rs7422195-*DR15* interaction term). The interaction *p*-value is the *p*-value associated with the rs7422195-*DR15* interaction term in this logistic regression model. A significant interaction *p*-value implies that the disease risk profile of *DR15* homozygous individuals across the possible rs7422195 genotypes (GG, AG, AA) is significantly different to the disease risk profile of *DR15* negative and heterozygous individuals across these rs7422195 genotypes.

### Haplotype construction and association analysis

Haplotypes were constructed and analysed for association using Haploview (v4.2; https://www.broadinstitute.org/scientific-community/science/programs/medical-and-population-genetics/haploview/haploview) using the confidence intervals algorithm. As default settings exclude MAF<0.05, all analyses were altered to include MAF>0.01. All blocks identified using this algorithm were tested for association within Haploview using chi-square tests.

### Cell subset purification

Immune cell subsets for RNA isolation were purified from Peripheral Blood Mononuclear Cells (PBMCs) using whole blood collected between 9am and 12pm. PBMCs were isolated from MS patients and controls using histopaque (Sigma-Aldrich, St Louis, MO) density gradient separation. CD4^+ve^ cells, CD8^+ve^ cells and B lymphocytes (CD19^+ve^) were purified using magnetic bead separation (positive selection) using Human Microbeads directed against the indicated cell surface markers as per the manufacturer’s instructions (Miltenyi Biotec, Macquarie Park, Australia). For CD14^+ve^ monocyte purification, a monocyte enrichment kit (Stemcell Technologies, Tullamarine, Australia) was used prior to selection of CD14^+ve^ cells with human CD14 Microbeads (Miltenyi Biotec, Macquarie Park, Australia) and magnetic bead purification. For Natural Killer (NK) cell (CD3^-ve^CD56^+ve^) purification, an NK enrichment kit (Stemcell Technologies, Tullamarine, Australia) was used as per manufacturer’s instructions prior to positive selection using human CD56 Microbeads (Miltenyi Biotec, Macquarie Park, Australia). Purified subsets were stored in RLT buffer (Qiagen) for subsequent RNA extraction. Purity of the subsets was determined by flow cytometry.

### Flow cytometric analysis

Purity of immune cell subtypes was assessed by labeling purified fractions with the antibodies CD4-PerCp (VIT4; Miltenyi Biotec, Macquarie Park, Australia), CD8-FITC (BW135/80; Miltenyi Biotec, Macquarie Park, Australia), CD20-PE (B lymphocytes; LT20; Miltenyi Biotec, Macquarie Park, Australia), CD14-PE (TUK4) and CD3-FITC (HIT3a; BD Pharmingen, Sparks, MD)/CD56-APC (AF12-7H3; Miltenyi Biotec, Macquarie Park, Australia) for NK cells. Flow cytometry of labeled PBMC and purified cell subsets was performed using a CyAn ADP analyzer (Beckman Coulter) and the data analysed using WEASEL (v3.0). Purified fractions for each of the cell subsets were only used in subsequent analyses if purity was 90% or greater.

In order to assess MERTK expression on the surface of monocytes, PBMCs were isolated from whole blood and monocytes identified using antibodies directed against the markers CD14-PE [TUK4 (Miltenyi Biotec, Macquarie Park, Australia)] and CD16-FITC [VEP13 (Miltenyi Biotec, Macquarie Park, Australia)] as previously described [44]. Cell surface MERTK protein was detected using human Mer APC-conjugated antibody [Clone #125518 (R&D Systems, Minneapolis, MN)] and compared with the appropriate isotype control [APC-conjugated Mouse IgG1, IS5-21F5 (Miltenyi Biotec, Macquarie Park, Australia)].

### Expression analysis of immune cell subsets

We extracted RNA from purified immune cell subsets and expression microarray hybridizations were performed using the WT Expression kit (Life Technologies, CA, USA), WT Terminal Labelling and Controls Kit (Affymetrix, CA, USA) and Affymetrix Human Gene_1.0ST arrays. The probed arrays were washed and stained using the GeneChip Hybridization Wash and Stain Kit (Affymetrix, CA, USA) and scanned using the GeneChip Scanner 3000. Images (.dat files) were processed using GeneChip Command Console (Affymetrix, CA, USA) and the CEL files generated were used for further analysis. Expression data was linearised by transformation to a log(2) scale and normalised using the removal of unwanted variation, 2-step (RUV-2) method[49]. Combined case and control datasets were used to identify *cis* eQTL associations across each cell type using an additive linear model. Statistically significant eQTL associations were defined as having p<0.05 following correction for multiple testing (ie. P<0.05 and FDR<0.05).

### *MERTK* expression analysis in monocytes using RNA sequencing (RNAseq)

Total RNA was isolated from purified monocytes using the Qiagen RNeasy minikit (Qiagen, Hilden, Germany) according to manufacturer's instructions. We then enriched the samples for mRNA using the Ambion polyA purist kit (ThermoFisher Scientific, Waltham, MA) according to manufacturer's instructions. Libraries for sequencing were prepared by the Australian Genome Research Facility (AGRF) from 200ng mRNA, with the mRNA from each individual ligated with a unique multiplex tag. Libraries were then pooled and divided across 3 lanes of the Illumina HiSeq sequencer (Illumina San Diego, CA), sequenced with 100bp single end reads and read quality assessed using FastQC (http://www.bioinformatics.bbsrc.ac.uk/projects/fastqc/). Untrimmed reads were aligned to human GRCh37/hg19 genome using Subjunc aligner within the Subread software package [50]. Sequencing data was summarized into reads per transcript using FeatureCounts [51] against the GENCODE 19 gene/isoform models for the human GRCh37/hg19 reference genome (July 2013 freeze) [52].

Normalisation and statistical analysis on the count data were executed using edgeR [53], keeping only those genes with >10 counts per million (CPM) in all samples of at least one of the two sample groups. Library sizes were between 83M and 91M reads and all samples were included in the analysis. Differential expression analysis was adjusted by edgeR for varying sequencing depths, and all normalisation factors were between 0.97 and 1.02 indicating that the data set was well-balanced. The data was scaled using trimmed mean of M-values (TMM) [54] and differentially expressed genes were called between the two sample groups if Benjamini–Hochberg false discovery rate (FDR) < 0.05. Annotation was added using the Ensembl human gene annotation using the R biomaRt package [55]. To visualise intron-exon boundaries and exon-exon junctions, mapped sequences were imported in the Integrative Genomics Viewer (IGV v.2.3.35; https://www.broadinstitute.org/software/igv/home).

### Reverse transcription PCR

Total RNA from monocytes was prepared as for expression analysis and reverse transcribed into cDNA using a Taqman Reverse Transcription kit according to manufacturer's instructions (Applied Biosystems, Scoresby, Australia). A reverse primer located within the putative alternative final exon 20 (GACAATGATTGGGATAGAAACC) was used in conjunction with either a forward primer for the canonical exon 18 (GAAATAGCTACGCGGGGAAT) or a forward primer in the canonical exon 19 (GTGTATATCATGGAAAAAGACAAGGAT). Amplification of cDNA was performed for 35 cycles with primer annealing at 60°C and products visualised on a 2% (w/v) agarose gel.

### Calculation of disease course

The prevalence of SPMS was determined using logistic regression analyses, whereby each patient was assigned a known duration of MS from diagnosis to time of collection, and at that same time was assessed as either RRMS or SPMS according to established criteria [14]. In conjunction with their genotype at rs7422195, regression analyses were performed to determine the whether the rate of change of SPMS (ie the % of SPMS cases/total cases at any given duration of MS) was different dependent on genotype at rs7422195.

### Statistical analysis

Gene expression data were analysed using either Student's *t*-test (for 2 groups) or one-way ANOVA (for >2 groups) followed by Tukey's multiple comparison correction (GraphPad PRISM v.6.0b; http://www.graphpad.com/scientific-software/prism/). Linear regression was used to analyse the relationship between *MERTK* gene expression and MERTK expression by monocytes (GraphPad PRISM v.6.0b). The proportion of monocytes expressing MERTK was performed using the Kruskal-Wallis test followed by Dunn's multiple comparison correction (GraphPad PRISM v.6.0b). All grouped expression data are plotted as Tukey box and whiskers. The relationship between *MERTK* genotype and Multiple Sclerosis severity was analysed using logistic regression (STATA v.12.1; http://www.stata.com/).

## Supporting Information

S1 Fig The rs7422195(A)-allele is associated with increased length of the TA_n_T_n_ repeat expansion in intron 1. The size of the amplified PCR product including the TA_n_T_n_ repeat within intron 1 was significantly increased in individuals homozygous for the rs7422195(A)-allele (p<0.0001 GG vs AA). For technical reasons only the shortest allele present in any individual was amplified.
(PDF)

S1 Table Hardy-Weinberg tests. (PDF)

S2 Table Apparent size of TA_n_T_n_ repeat expansion in tested samples. (PDF)

S3 Table Analysis of linkage between rs13414207 and the intron 4 AluYf4 insertion in two populations. (PDF)
