# Supplementary figures and images for: Common and Low Frequency Variants in MERTK Are Independently Associated with Multiple Sclerosis Susceptibility with Discordant Association Dependent upon HLA-DRB1*15:01 Status
Source: PLoS Genet. 2016 Mar 18;12(3):e1005853. doi: 10.1371/journal.pgen.1005853 (PMC4798184; doi:10.1371/journal.pgen.1005853)

Supplemental Figure S1

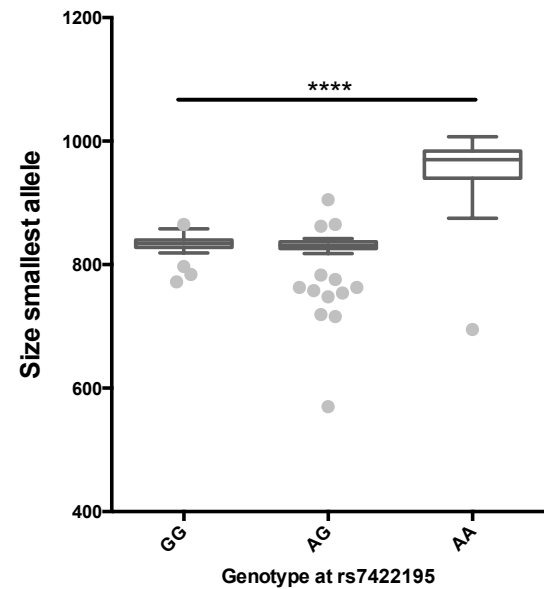

Supplement: S1 Fig — The size of the amplified PCR product including the TAnTn repeat within intron 1 was significantly increased in individuals homozygous for the rs7422195(A)-allele (p<0.0001 GG vs AA). For technical reasons only the shortest allele present in any individual was amplified. (PDF) [file pgen.1005853.s001.pdf]
