# Supplementary material for: Common and Low Frequency Variants in MERTK Are Independently Associated with Multiple Sclerosis Susceptibility with Discordant Association Dependent upon HLA-DRB1*15:01 Status
Source: PLoS Genet. 2016 Mar 18;12(3):e1005853. doi: 10.1371/journal.pgen.1005853 (PMC4798184; doi:10.1371/journal.pgen.1005853)
Supplement: S1 Table — (PDF) [file pgen.1005853.s002.pdf]

Table S1: Hardy-Weinberg Tests

Variant	Chr.[a]	Location[b]	All samples			Cases			Controls		
			Obs[c] Het	Exp[d] Het	*p*-value	Obs Het	Exp Het	*p*-value	Obs Het	Exp Het	*p*-value
Variants tested in fine-mapping study											
rs1063	2	18026245	0.2589	0.2641	0.09887	0.2581	0.2621	0.3847	0.2596	0.266	0.165
rs1109465	2	26606154	0.1284	0.1286	0.9248	0.1368	0.1343	0.3598	0.1207	0.1233	0.2191
rs12466022	2	43359061	0.392	0.3911	0.8771	0.3866	0.379	0.2675	0.3969	0.4016	0.4792
rs7595037	2	68647095	0.4919	0.4929	0.8633	0.4917	0.4909	0.943	0.4921	0.4945	0.7859
1kgp_chr2_0158.SNP	2	112358325	0.4959	0.4961	0.9805	0.4943	0.4963	0.8321	0.4973	0.4959	0.8926
1kgp_chr2_1082.SNP	2	112358386	0.03595	0.03643	0.2993	0.03523	0.03579	0.2925	0.03661	0.03702	0.3622
1kgp_chr2_0160.SNP	2	112358829	0.4992	0.4945	0.4507	0.515	0.4956	0.0324	0.4847	0.4933	0.3116
1kgp_chr2_0170.SNP	2	112360602	0.3831	0.3839	0.8747	0.381	0.3844	0.6162	0.3851	0.3833	0.827
1kgp_chr2_1086.SNP	2	112361648	0.005845	0.005828	1	0.005819	0.005802	1	0.005869	0.005852	1
1kgp_chr2_1087.SNP	2	112361890	0.09358	0.09343	1	0.09289	0.09301	0.8509	0.09421	0.09382	1
1kgp_chr2_1092.SNP	2	112367731	0	0	1	0	0	1	0	0	1
1kgp_chr2_1093.SNP	2	112367876	0.1793	0.1785	0.7347	0.1728	0.1715	0.759	0.1853	0.1848	1
1kgp_chr2_0191.SNP	2	112368535	0.1264	0.127	0.6346	0.1304	0.1325	0.3549	0.1227	0.122	0.8907
1kgp_chr2_1760.SNP	2	112368729	0	0	1	0	0	1	0	0	1
1kgp_chr2_1095.SNP	2	112369363	0.3331	0.3346	0.7176	0.3299	0.3296	1	0.3361	0.3391	0.6212
1kgp_chr2_1096.SNP	2	112369671	0	0	1	0	0	1	0	0	1
1kgp_chr2_0192.SNP	2	112370186	0.08695	0.08716	0.7815	0.09186	0.09428	0.1369	0.08247	0.08061	0.2127
1kgp_chr2_0193.SNP	2	112370273	0.308	0.3076	0.9683	0.3045	0.306	0.7727	0.3111	0.3091	0.7426
1kgp_chr2_1098.SNP	2	112370675	0.02322	0.02295	1	0.02111	0.02088	1	0.02515	0.02484	1
1kgp_chr2_0194.SNP	2	112371433	0.235	0.2315	0.2294	0.2371	0.2341	0.5017	0.2332	0.2291	0.3074
1kgp_chr2_1099.SNP	2	112371713	0.4227	0.4238	0.8413	0.4262	0.4215	0.5604	0.4194	0.4259	0.3649
1kgp_chr2_1100.SNP	2	112371837	0.0126	0.01252	1	0.01321	0.01312	1	0.01204	0.01197	1
1kgp_chr2_1101.SNP	2	112371941	0.03629	0.03591	0.7299	0.03591	0.03527	0.626	0.03663	0.0365	1
1kgp_chr2_1174.SNP	2	112403459	0.4221	0.4242	0.6893	0.4264	0.4211	0.5053	0.4183	0.4269	0.2238
rs6708131	2	112445659	0.4894	0.4892	1	0.4901	0.4877	0.798	0.4887	0.4906	0.835
rs6733876	2	112447366	0.1059	0.1061	0.9088	0.1112	0.1121	0.637	0.1011	0.1005	0.8679
1kgp_chr2_0355.SNP	2	112468399	0.4738	0.47	0.5199	0.4865	0.4738	0.1298	0.4622	0.4664	0.5905
1kgp_chr2_1285.SNP	2	112471351	0.4267	0.4262	0.9319	0.4319	0.4232	0.2627	0.422	0.4289	0.3473
1kgp_chr2_1286.SNP	2	112471414	0.4238	0.425	0.8198	0.4296	0.4222	0.3401	0.4186	0.4275	0.2102
1kgp_chr2_0389.SNP	2	112482444	0.4765	0.4708	0.3283	0.4901	0.4745	0.06441	0.4641	0.4673	0.6935
rs17040773	2	112500035	0.3755	0.3686	0.1339	0.3829	0.3741	0.2002	0.3686	0.3635	0.4263
1kgp_chr2_1342.SNP	2	112503333	0	0	1	0	0	1	0	0	1
1kgp_chr2_0441.SNP	2	112503461	0.06393	0.06489	0.194	0.06742	0.06971	0.07249	0.06073	0.06046	1
1kgp_chr2_0442.SNP	2	112503686	0.1386	0.14	0.388	0.1451	0.1512	0.02728	0.1326	0.1296	0.1965
1kgp_chr2_0443.SNP	2	112504276	0.1133	0.1183	0.001217	0.1196	0.1294	9.11E-05	0.1076	0.1079	0.7547
1kgp_chr2_0445.SNP	2	112504566	0.1383	0.1395	0.4877	0.1448	0.1504	0.04657	0.1325	0.1295	0.1965
1kgp_chr2_0446.SNP	2	112504851	0.1377	0.139	0.4336	0.144	0.1497	0.03467	0.132	0.1291	0.1966

1kgp_chr2_1346.SNP	2	112505086	0	0	1	0	0	1	0	0	1
1kgp_chr2_1347.SNP	2	112505652	0.00322	0.003215	1	0.002143	0.002141	1	0.004206	0.004198	1
1kgp_chr2_1804.SNP	2	112505721	0.0004388	0.0004387	1	0.0006126	0.0006124	1	0.00028	0.0002799	1
1kgp_chr2_0449.SNP	2	112505852	0.1382	0.1397	0.3864	0.1448	0.1504	0.0466	0.1322	0.1297	0.3027
1kgp_chr2_0452.SNP	2	112506327	0.03902	0.03826	0.1161	0.04167	0.0408	0.4028	0.03661	0.03594	0.6326
1kgp_chr2_1806.SNP	2	112506527	0	0	1	0	0	1	0	0	1
1kgp_chr2_1350.SNP	2	112507033	0.4213	0.4177	0.4871	0.4303	0.4153	0.04297	0.413	0.4198	0.3386
rs1402939	2	112521897	0.4965	0.4897	0.254	0.4943	0.4908	0.6938	0.4986	0.4886	0.2423
1kgp_chr2_0482.SNP	2	112524749	0.4569	0.4415	0.004186	0.4684	0.4408	0.0003882	0.4462	0.4421	0.5938
1kgp_chr2_0483.SNP	2	112525307	0.3795	0.3802	0.8737	0.3725	0.3704	0.7767	0.3858	0.3888	0.6364
1kgp_chr2_0484.SNP	2	112525690	0.4023	0.4027	0.9521	0.3907	0.3928	0.7552	0.413	0.4112	0.8388
1kgp_chr2_0485.SNP	2	112526036	0.0613	0.0619	0.4281	0.06583	0.06823	0.06352	0.05711	0.05601	0.3689
1kgp_chr2_1374.SNP	2	112526422	0.4204	0.4179	0.6433	0.4287	0.4157	0.07692	0.4129	0.4199	0.3194
1kgp_chr2_0489.SNP	2	112527493	0.4458	0.442	0.4939	0.4571	0.4426	0.06875	0.4355	0.4414	0.426
1kgp_chr2_0490.SNP	2	112527553	0.4456	0.4424	0.5659	0.4564	0.4435	0.1058	0.4357	0.4414	0.4482
1kgp_chr2_0491.SNP	2	112528327	0.3176	0.3238	0.114	0.3201	0.3227	0.6612	0.3153	0.3249	0.07836
1kgp_chr2_1377.SNP	2	112529327	0.4219	0.4193	0.6217	0.4289	0.4165	0.09851	0.4156	0.4218	0.3804
1kgp_chr2_1379.SNP	2	112529537	0.4442	0.4425	0.763	0.4546	0.4429	0.1415	0.4348	0.4421	0.3249
1kgp_chr2_0494.SNP	2	112529634	0.4441	0.4424	0.7631	0.4543	0.4426	0.1418	0.4348	0.4421	0.3247
rs10190220	2	112583824	0.1377	0.1315	1.95E-05	0.146	0.14	0.01203	0.1301	0.1236	0.00058
rs11682030	2	112585876	0.4363	0.4476	0.04003	0.4404	0.4421	0.8418	0.4326	0.4525	0.009427
rs17040720	2	112622593	0.2796	0.2817	0.5479	0.2851	0.2807	0.4177	0.2746	0.2827	0.09715
rs4848821	2	112626773	0.189	0.187	0.4377	0.1849	0.1811	0.2871	0.1927	0.1924	1
rs17017209	2	112636718	0.02381	0.02353	1	0.02417	0.02387	1	0.02349	0.02321	1
rs17835589	2	112641149	0.4088	0.4012	0.1236	0.4087	0.4025	0.3836	0.4089	0.4001	0.1947
rs3814026	2	112641666	0.4971	0.4987	0.7894	0.4998	0.4981	0.8602	0.4947	0.4992	0.5917
rs13016143	2	112649472	0.4608	0.4576	0.5783	0.4643	0.457	0.3774	0.4577	0.4581	0.9708
rs12329119	2	112649794	0.359	0.3567	0.6348	0.3536	0.3531	0.9605	0.3639	0.36	0.5457
rs34787974	2	112650905	0.3636	0.3598	0.4005	0.3623	0.3571	0.4325	0.3648	0.3622	0.7118
rs7604403	2	112656652	0.4204	0.4227	0.6658	0.4213	0.4192	0.8004	0.4197	0.4258	0.4077
rs1516629	2	112658779	0.3633	0.3578	0.2114	0.3513	0.3446	0.2864	0.3742	0.3693	0.4419
rs869016	2	112659110	0.4678	0.4629	0.3879	0.4792	0.4666	0.1325	0.4574	0.4593	0.7987
rs13006203	2	112661150	0.4261	0.4243	0.7317	0.4271	0.421	0.4286	0.4252	0.4272	0.7834
rs10209260	2	112662407	0.08755	0.08824	0.4923	0.09096	0.09348	0.1308	0.08443	0.08342	0.6859
rs7595212	2	112662767	0.4233	0.4241	0.8642	0.4248	0.4211	0.6473	0.4219	0.4269	0.4809
rs17174697	2	112662914	0.4197	0.4214	0.7497	0.42	0.4173	0.7351	0.4194	0.425	0.449
rs12989536	2	112663919	0.1752	0.1716	0.0903	0.1747	0.1744	1	0.1756	0.1689	0.01702
rs10496439	2	112664631	0.4242	0.4234	0.909	0.4263	0.4203	0.4284	0.4222	0.4262	0.5826
rs10496440	2	112664999	0.1436	0.1432	0.9327	0.1433	0.1445	0.6274	0.1439	0.1421	0.5553
rs17174870	2	112665201	0.3638	0.3595	0.345	0.3498	0.3454	0.4777	0.3766	0.3719	0.4703
rs17779482	2	112665677	0.4241	0.4239	0.9772	0.4266	0.4208	0.4538	0.4219	0.4267	0.5052
rs11673729	2	112665961	0.4246	0.4242	0.9545	0.4265	0.4214	0.5065	0.4229	0.4267	0.5832

rs7349221	2	112668341	0.4241	0.4244	0.9545	0.4265	0.4213	0.5059	0.4219	0.4272	0.4571
rs4848861	2	112668835	0.424	0.4236	0.9544	0.4259	0.4205	0.4792	0.4222	0.4263	0.5825
rs7568632	2	112669950	0.3641	0.3577	0.1459	0.3519	0.3447	0.2428	0.3751	0.369	0.3412
rs1400321	2	112670046	0.3714	0.3617	0.03124	0.3575	0.3477	0.1174	0.3842	0.3741	0.1144
rs1400322	2	112670185	0.5036	0.4969	0.2719	0.5124	0.4988	0.1292	0.4957	0.4943	0.8922
rs1400323	2	112670461	0.5074	0.4969	0.08309	0.5188	0.4987	0.02423	0.497	0.4944	0.7602
rs4264571	2	112674149	0.4234	0.4246	0.8188	0.4239	0.4214	0.7704	0.4229	0.4274	0.5287
rs1516639	2	112675085	0.5065	0.4967	0.1072	0.519	0.4988	0.02214	0.4952	0.494	0.8921
rs908518	2	112677626	0.473	0.4707	0.6994	0.4868	0.4746	0.1496	0.4604	0.4668	0.4091
rs867311	2	112677970	0.3573	0.3566	0.8919	0.3453	0.3433	0.7593	0.3681	0.3682	1
rs1996328	2	112680741	0.4743	0.47	0.4553	0.4879	0.4739	0.09622	0.4619	0.4661	0.5904
rs1400325	2	112680934	0.364	0.358	0.1661	0.3529	0.3456	0.2439	0.3741	0.3688	0.4141
rs1400324	2	112681220	0.365	0.3573	0.0782	0.3547	0.3449	0.1153	0.3745	0.3682	0.3175
rs4848874	2	112681500	0.425	0.4264	0.7982	0.43	0.4238	0.4317	0.4204	0.4288	0.2571
rs11687451	2	112682561	0.4238	0.4249	0.842	0.4284	0.4217	0.3834	0.4196	0.4277	0.2567
rs4468823	2	112684371	0.4741	0.4702	0.5036	0.4876	0.4741	0.1121	0.4618	0.4665	0.5662
rs6711987	2	112690175	0.4785	0.4974	0.002698	0.491	0.4989	0.4001	0.4669	0.4953	0.001121
rs4848229	2	112692650	0.4236	0.4254	0.7119	0.4284	0.4224	0.4314	0.4192	0.4282	0.2114
rs10207820	2	112699350	0.4747	0.4695	0.3663	0.4892	0.4734	0.05881	0.4615	0.4657	0.5895
rs884448	2	112700328	0.5051	0.4965	0.1575	0.5169	0.4986	0.03807	0.4943	0.4938	0.973
rs11683694	2	112702647	0.4249	0.4256	0.8869	0.4294	0.4226	0.3834	0.4208	0.4283	0.2917
rs11692445	2	112704033	0.4247	0.425	0.9546	0.429	0.4222	0.3838	0.4207	0.4274	0.3475
rs3761700	2	112705185	0.5054	0.4965	0.1425	0.5182	0.4985	0.02632	0.4937	0.4938	1
rs3761699	2	112705290	0.424	0.426	0.6911	0.4284	0.4233	0.5086	0.4199	0.4284	0.2413
rs1113419	2	112707483	0.363	0.3569	0.1649	0.3508	0.3437	0.2624	0.3741	0.3684	0.3885
rs4848901	2	112710828	0.5054	0.4964	0.1366	0.5174	0.4984	0.03191	0.4945	0.4938	0.9459
rs17175275	2	112712329	0.3645	0.3579	0.137	0.3537	0.3454	0.1872	0.3744	0.3689	0.3892
rs11674883	2	112713069	0.4243	0.426	0.7547	0.4287	0.4231	0.4816	0.4203	0.4286	0.2573
rs10187656	2	112716047	0.08898	0.0898	0.419	0.09216	0.09511	0.09334	0.08608	0.08493	0.5541
rs7573344	2	112716317	0	0	1	0	0	1	0	0	1
rs1122970	2	112716697	0.4245	0.4255	0.8423	0.4284	0.4225	0.4556	0.421	0.4283	0.31
rs1546636	2	112722418	0.05862	0.05882	0.6807	0.06459	0.06536	0.4209	0.05316	0.05281	1
rs6541971	2	112722490	0.4754	0.47	0.3531	0.4875	0.4735	0.09563	0.4642	0.4665	0.7736
rs4848908	2	112723117	0.3032	0.3032	1	0.305	0.3055	0.9087	0.3015	0.3011	1
rs7579906	2	112723366	0.4745	0.4697	0.4094	0.4877	0.4736	0.09554	0.4625	0.4658	0.6664
rs10185747	2	112723865	0.08806	0.08924	0.2762	0.09158	0.09514	0.04091	0.08485	0.08381	0.6868
rs10084408	2	112724421	0.0891	0.09017	0.3456	0.09269	0.09613	0.0454	0.08583	0.08469	0.5539
rs2271701	2	112731173	0.4236	0.4263	0.5896	0.4279	0.4233	0.5623	0.4196	0.429	0.1976
rs1607059	2	112734512	0.4753	0.4696	0.3271	0.4882	0.4732	0.07532	0.4635	0.4661	0.7465
rs10166564	2	112736723	0.4748	0.4692	0.3267	0.4869	0.4726	0.0877	0.4639	0.4659	0.8015
rs10199616	2	112737185	0.4733	0.4696	0.5364	0.4842	0.4732	0.1956	0.4633	0.4661	0.7194
rs6725192	2	112738562	0.4739	0.47	0.5029	0.4857	0.4735	0.1483	0.4632	0.4666	0.6671

rs17175626	2	112740597	0.3646	0.3544	0.01827	0.3528	0.3413	0.05707	0.3752	0.3658	0.1307
rs10180345	2	112741293	0.474	0.4695	0.439	0.4857	0.4733	0.1387	0.4632	0.4658	0.7462
rs13020304	2	112741535	0.4253	0.4256	0.9547	0.4305	0.4226	0.3002	0.4206	0.4282	0.2914
rs1996325	2	112742075	0.4722	0.4703	0.738	0.4842	0.4738	0.2222	0.4614	0.4668	0.496
rs4848923	2	112742451	0.4753	0.4698	0.3403	0.4885	0.4735	0.07535	0.4633	0.4662	0.7195
rs11675946	2	112743033	0.4469	0.4476	0.8925	0.4572	0.4477	0.2408	0.4374	0.4475	0.1782
rs11676037	2	112743385	0.4719	0.469	0.6407	0.4871	0.4726	0.08664	0.458	0.4656	0.3294
rs1516628	2	112743800	0.4748	0.4697	0.3811	0.4876	0.4734	0.09575	0.4632	0.4662	0.7196
rs1554214	2	112743917	0.4765	0.469	0.1946	0.4896	0.4732	0.05277	0.4646	0.4649	0.9712
rs1554215	2	112744098	0.4745	0.47	0.4394	0.4868	0.4744	0.1495	0.4633	0.4657	0.7734
rs11682629	2	112744299	0.424	0.4253	0.798	0.4289	0.4224	0.3845	0.4195	0.428	0.241
rs1996327	2	112744727	0.4736	0.4681	0.3364	0.4882	0.4715	0.04791	0.4602	0.4647	0.5624
rs13394651	2	112745114	0.4763	0.4698	0.2672	0.4898	0.4737	0.05852	0.464	0.466	0.8014
rs870761	2	112745290	0.4241	0.4249	0.8867	0.4292	0.4222	0.3612	0.4194	0.4273	0.2723
rs13385764	2	112746891	0.4759	0.4703	0.3275	0.4895	0.474	0.06409	0.4635	0.4666	0.6931
rs13401834	2	112747331	0.08755	0.08797	0.6789	0.09213	0.09398	0.2603	0.08336	0.08244	0.6839
rs11123073	2	112747653	0.4737	0.47	0.52	0.4868	0.4736	0.1204	0.4618	0.4665	0.5423
rs13414207	2	112748053	0.0739	0.0759	0.0493	0.08908	0.09308	0.02141	0.0599	0.05973	1
rs11685190	2	112748704	0.4248	0.4257	0.8647	0.4297	0.4227	0.3623	0.4204	0.4283	0.2741
rs7571009	2	112749511	0.4724	0.4698	0.6613	0.4857	0.4734	0.1485	0.4603	0.4664	0.4298
rs7571120	2	112749594	0.4736	0.472	0.7951	0.4858	0.4756	0.2324	0.4625	0.4685	0.4478
rs7592909	2	112752142	0.4735	0.4703	0.5894	0.4858	0.4742	0.1722	0.4622	0.4665	0.5908
rs6739294	2	112752880	0.4759	0.4703	0.3402	0.4898	0.4742	0.0641	0.4632	0.4665	0.6928
rs6726639	2	112753097	0.4749	0.4699	0.3953	0.4879	0.4737	0.09582	0.4632	0.4663	0.693
rs11683421	2	112753230	0.4768	0.471	0.3151	0.4903	0.4748	0.06974	0.4645	0.4673	0.7198
rs10173553	2	112753883	0.4738	0.4699	0.5032	0.4867	0.4736	0.1204	0.4621	0.4663	0.5904
rs13003919	2	112753950	0.4243	0.4246	0.9773	0.4294	0.4215	0.2988	0.4197	0.4273	0.2903
rs4848238	2	112754720	0.4233	0.426	0.6093	0.4287	0.4235	0.5084	0.4184	0.4282	0.1714
rs3811636	2	112755209	0.4739	0.47	0.5033	0.4867	0.4738	0.1297	0.4623	0.4662	0.6154
rs10183215	2	112756645	0.08846	0.0896	0.2796	0.09133	0.09492	0.03999	0.08585	0.08472	0.5539
rs4519530	2	112759182	0.4752	0.4699	0.367	0.4889	0.4739	0.07553	0.4627	0.4661	0.6665
rs4528767	2	112759864	0.3639	0.358	0.1769	0.3536	0.3459	0.2065	0.3733	0.3686	0.468
rs13430221	2	112760875	0.09714	0.09691	1	0.09476	0.0969	0.2033	0.09933	0.09692	0.1659
rs4303721	2	112761034	0.476	0.4714	0.4259	0.4891	0.475	0.09691	0.4641	0.4678	0.6417
rs4278932	2	112761310	0.3643	0.3573	0.1116	0.3544	0.345	0.128	0.3734	0.3681	0.4135
rs4848933	2	112761653	0.4757	0.4714	0.4721	0.4888	0.475	0.1041	0.4638	0.468	0.5919
rs7580261	2	112762623	0.251	0.2481	0.3543	0.2531	0.2501	0.5286	0.249	0.2462	0.5412
rs13432863	2	112763269	0.08885	0.08968	0.4179	0.09216	0.09511	0.09334	0.08583	0.08469	0.5539
rs10195619	2	112765562	0.4711	0.472	0.8773	0.4837	0.4738	0.2505	0.4596	0.4703	0.1742
rs11884641	2	112767406	0.364	0.3582	0.1881	0.3544	0.346	0.1724	0.3728	0.3689	0.5558
rs7563113	2	112768177	0.3643	0.3582	0.1665	0.3543	0.3463	0.1894	0.3734	0.3687	0.468
rs10181117	2	112768439	0.0843	0.08533	0.3173	0.08714	0.09009	0.07512	0.08171	0.08096	0.8342

rs12469210	2	112768991	0.3642	0.3575	0.1277	0.3546	0.3455	0.1417	0.373	0.368	0.4394
rs11683409	2	112770134	0.478	0.4716	0.2693	0.4906	0.4742	0.04988	0.4665	0.4691	0.748
rs10179948	2	112770821	0.09928	0.0991	1	0.1017	0.1026	0.6063	0.09707	0.09591	0.5995
rs13387346	2	112773057	0.06413	0.0648	0.3501	0.06793	0.07018	0.07624	0.06065	0.05986	0.7755
rs6710733	2	112773500	0.3648	0.3569	0.06716	0.3549	0.3447	0.09368	0.3739	0.3676	0.3168
rs6711146	2	112773834	0.4809	0.4758	0.387	0.4951	0.4785	0.05209	0.4679	0.4731	0.5243
rs10211152	2	112775509	0.06413	0.0648	0.3501	0.06793	0.07018	0.07624	0.06065	0.05986	0.7755
rs10168067	2	112777232	0.06384	0.06426	0.5693	0.06734	0.06906	0.1943	0.06065	0.05986	0.7755
rs6712080	2	112777441	0.3635	0.3577	0.1873	0.3531	0.3457	0.2246	0.373	0.3682	0.4673
rs11683819	2	112778441	0.4794	0.4757	0.524	0.4907	0.4786	0.1518	0.4692	0.4729	0.6456
rs6737989	2	112779449	0.365	0.3578	0.1047	0.3551	0.3454	0.1162	0.374	0.3688	0.4142
rs12989808	2	112779734	0.1844	0.1988	2.16E-08	0.1836	0.2053	2.62E-08	0.1852	0.1928	0.02322
rs13419523	2	112781917	0.1378	0.1394	0.3848	0.1437	0.15	0.02555	0.1325	0.1295	0.1965
rs6730521	2	112783957	0.3643	0.3579	0.1453	0.3537	0.3451	0.1701	0.3739	0.3691	0.4668
rs6541998	2	112785237	0.48	0.4753	0.43	0.4932	0.4782	0.07858	0.468	0.4726	0.5711
rs6723289	2	112785640	0.0639	0.06543	0.06139	0.06699	0.0699	0.0372	0.06105	0.06131	0.7798
rs10180086	2	112785891	0.06446	0.06566	0.1372	0.06793	0.07074	0.04031	0.06129	0.06099	1
rs2230516	2	112786034	0.004062	0.004054	1	0.005361	0.005347	1	0.002877	0.002873	1
rs6710189	2	112789792	0.38	0.3805	0.8988	0.3874	0.3946	0.3071	0.3733	0.3669	0.316
rs4848971	2	112790446	0.4196	0.4164	0.5405	0.4276	0.4139	0.06185	0.4122	0.4186	0.3565
rs10211634	2	112791122	0.06008	0.06075	0.3188	0.06428	0.06678	0.05687	0.05623	0.05518	0.3688
rs10169022	2	112792656	0.4436	0.4409	0.6204	0.4568	0.4423	0.06817	0.4315	0.4396	0.2844
rs11684476	2	112793981	0.4452	0.4423	0.6033	0.4562	0.4431	0.09711	0.4351	0.4416	0.3834
rs11897014	2	112795908	0.4455	0.4322	0.0122	0.4514	0.4312	0.008652	0.4401	0.4332	0.3526
rs9631022	2	112801179	0.06166	0.06195	0.6934	0.06614	0.06794	0.1218	0.05757	0.05644	0.3703
rs6748256	2	112803874	0.4954	0.4993	0.5282	0.5023	0.4997	0.7791	0.4891	0.4986	0.2533
rs13001162	2	112805997	0.4206	0.4176	0.5623	0.4303	0.4157	0.04754	0.4118	0.4193	0.2813
rs6713344	2	112814045	0.3951	0.393	0.6883	0.381	0.381	1	0.4079	0.4034	0.5327
rs4848978	2	112816047	0.4036	0.4033	0.9761	0.392	0.3936	0.824	0.4141	0.4117	0.7452
rs4848979	2	112816236	0.4294	0.4362	0.202	0.4391	0.4358	0.6878	0.4207	0.4366	0.02899
rs4848980	2	112816348	0.4195	0.4177	0.7496	0.4279	0.4153	0.09113	0.4119	0.4199	0.2634
rs10206255	2	112834876	0.03977	0.0401	0.5353	0.04196	0.04342	0.07433	0.03777	0.03706	0.638
rs10182333	2	112841856	0.4982	0.4959	0.7146	0.5008	0.4951	0.5248	0.4959	0.4966	0.9463
rs2067928	2	112850581	0.2058	0.2034	0.3419	0.2065	0.2057	0.9322	0.2053	0.2013	0.2795
rs9653422	2	112857642	0.5064	0.5	0.2978	0.5138	0.5	0.1232	0.4996	0.5	0.9733
rs4848272	2	112858122	0.477	0.4786	0.7789	0.4813	0.4784	0.7685	0.473	0.4789	0.4782
rs7583755	2	112858485	0.1719	0.1716	0.9437	0.1746	0.1754	0.7648	0.1695	0.168	0.6902
rs954521	2	112866558	0.1354	0.1343	0.5295	0.1447	0.1441	0.9036	0.1269	0.1252	0.5034
rs7581849	2	112870730	0.4731	0.4678	0.351	0.4725	0.4653	0.3861	0.4737	0.47	0.6429
rs10172310	2	112883781	0.04126	0.04153	0.551	0.04388	0.04526	0.09433	0.03887	0.03811	0.6447
rs10166396	2	112891822	0.1185	0.12	0.3133	0.1276	0.1327	0.03407	0.1103	0.1082	0.3503
rs10202871	2	112898848	0	0	1	0	0	1	0	0	1

rs7559210	2	112912699	0.05846	0.06005	0.04028	0.06618	0.06798	0.1221	0.05143	0.05275	0.1874
rs2312695	2	112920849	0.04644	0.04731	0.1232	0.05017	0.05065	0.4827	0.04304	0.04425	0.1079
rs12620229	2	112921956	0.492	0.489	0.6384	0.5031	0.4896	0.1241	0.4818	0.4885	0.4112
rs10200201	2	112928815	0.4764	0.4965	0.0009267	0.4821	0.4954	0.1272	0.4712	0.4974	0.001783
rs10191773	2	112930064	0.3307	0.3303	0.9708	0.3359	0.3306	0.3969	0.3259	0.33	0.4466
rs7557904	2	112931936	0.2408	0.2407	1	0.2409	0.245	0.352	0.2407	0.2368	0.3573
rs7600843	2	112939042	0.5048	0.4998	0.4237	0.5222	0.4999	0.01263	0.4889	0.4997	0.2029
rs11885036	2	112952799	0.1335	0.1339	0.7855	0.1414	0.1445	0.2232	0.1262	0.1242	0.4154
rs10183121	2	112953542	0.505	0.497	0.1879	0.5012	0.4969	0.6458	0.5084	0.4971	0.1779
rs17041977	2	112959668	0.06768	0.0684	0.3726	0.06792	0.06847	0.6035	0.06747	0.06834	0.454
rs11685462	2	112971931	0.2615	0.2579	0.2608	0.2586	0.2539	0.302	0.2641	0.2615	0.609
rs1343666	2	112986955	0.4034	0.4004	0.5456	0.3987	0.3918	0.3258	0.4077	0.4079	1
rs34239351	2	112997066	0.02124	0.02101	1	0.01622	0.01609	1	0.02584	0.02551	1
rs11674953	2	113014080	0.475	0.4729	0.7389	0.4832	0.4738	0.2669	0.4675	0.4721	0.5694
rs6542045	2	113015512	0.4968	0.498	0.8631	0.4946	0.4977	0.7199	0.4989	0.4982	0.973
rs4471897	2	113016478	0.03844	0.03882	0.344	0.03735	0.03725	1	0.03943	0.04026	0.1886
rs6705046	2	113017065	0.06105	0.06221	0.1197	0.06359	0.06502	0.1812	0.05874	0.05964	0.3883
rs13001618	2	113036839	0	0	1	0	0	1	0	0	1
rs10165123	2	113041461	0.08854	0.08888	0.6837	0.08469	0.08614	0.3075	0.09205	0.09137	0.854
rs4430968	2	113053061	0.4582	0.4622	0.4908	0.4633	0.4642	0.9093	0.4534	0.4602	0.3946
rs6760417	2	113072762	0	0	1	0	0	1	0	0	1
rs7565648	2	113094694	0.0001468	0.0001468	1	0	0	1	0.0002814	0.0002813	1
rs13407095	2	113112005	0.111	0.1122	0.3855	0.1152	0.1178	0.2295	0.1072	0.107	1
rs13017673	2	113214648	0.2034	0.2	0.1642	0.205	0.2051	0.9321	0.202	0.1953	0.03976
rs7581524	2	113234316	0.413	0.4072	0.2464	0.408	0.4082	1	0.4176	0.4063	0.09961
rs3961919	2	113243321	0.4902	0.4876	0.6728	0.4934	0.4861	0.4066	0.4872	0.4889	0.8372
rs6707618	2	113243799	0.2692	0.2637	0.0816	0.2651	0.2605	0.3471	0.2731	0.2665	0.1494
rs1568120	2	135406890	0.4757	0.4893	0.02153	0.4727	0.4928	0.02094	0.4784	0.4856	0.389
rs10164986	2	135468385	0.273	0.2773	0.2056	0.2794	0.282	0.6188	0.2672	0.2729	0.2198
rs7571217	2	192327964	0.06727	0.06665	0.5844	0.06865	0.06686	0.1844	0.06601	0.06645	0.6115
rs281783	2	200751582	0.3053	0.311	0.1387	0.2997	0.3037	0.4532	0.3105	0.3176	0.1868
rs1831024	2	215858828	0.1671	0.1702	0.1356	0.1674	0.1735	0.0544	0.1669	0.1673	0.842
rs718686	2	218385952	0.3347	0.3321	0.5357	0.3368	0.3308	0.3151	0.3328	0.3333	0.9199
rs10201872	2	231106724	0.3153	0.3093	0.1174	0.336	0.3242	0.04014	0.2964	0.2951	0.8205
rs9271366	6	32586854	0.3681	0.3668	0.7898	0.4857	0.449	3.781E-06	0.2617	0.2589	0.5575
Variants tested in second round of association testing											
rs9271366	6	32586854	0.3685	0.3637	0.5125	0.4728	0.4364	0.001335	0.2643	0.2637	1
rs115416402	2	112645419	0.09274	0.09207	0.8434	0.08908	0.08877	1	0.09639	0.09536	1
rs115531771	2	112648710	0.0944	0.09296	0.5533	0.09024	0.0886	0.7676	0.09854	0.09727	0.7941
rs72823471	2	112655396	0.06308	0.06359	0.5629	0.05544	0.05643	0.3578	0.07071	0.07069	1
rs115821982	2	112656392	0.08788	0.09075	0.1006	0.07925	0.08472	0.02285	0.09656	0.09676	0.79

rs72409080	2	112660712	0.08896	0.09229	0.06928	0.08161	0.08805	0.01143	0.09632	0.0965	0.7908
rs17174870	2	112665201	0.3677	0.3581	0.1517	0.3589	0.3505	0.3751	0.3764	0.3656	0.2868
rs150406081	2	112667020	0.03732	0.03727	1	0.03336	0.03409	0.3624	0.04128	0.04043	1
rs4271775	2	112675149	0.09008	0.09336	0.07189	0.08193	0.08838	0.01169	0.09831	0.09835	1
rs56264210	2	112684419	0.08801	0.09085	0.1017	0.08094	0.08622	0.02806	0.09514	0.09549	0.785
rs72823495	2	112688481	0.0877	0.09057	0.1001	0.07879	0.08432	0.02143	0.09658	0.09675	0.7914
rs72823500	2	112695386	0.08976	0.09299	0.07248	0.08088	0.08738	0.01044	0.0986	0.09853	1
rs10165614	2	112698460	0.08994	0.09314	0.07352	0.08211	0.08849	0.0123	0.09774	0.09775	1
rs13404771	2	112702284	0.08905	0.09177	0.1086	0.07965	0.08628	0.00882	0.09846	0.09723	1
rs10171303	2	112705427	0.08899	0.09292	0.02846	0.08104	0.08877	0.003652	0.09693	0.09704	0.7934
rs9808061	2	112706759	0.08966	0.09294	0.07106	0.08121	0.08772	0.01069	0.09812	0.09814	1
rs13388623	2	112708321	0.08881	0.09214	0.06904	0.08139	0.08782	0.01125	0.09626	0.09644	0.7907
rs143306687	2	112710056	0.08929	0.092	0.1094	0.07992	0.08533	0.02465	0.09866	0.09862	1
rs13393166	2	112710205	0.08973	0.09296	0.07242	0.0815	0.08794	0.01134	0.09793	0.09793	1
NOVEL SNP	2	112718074	0.08434	0.102	1.01E-13	0.07609	0.09772	1.68E-10	0.09256	0.1063	3.11E-05
rs28529268	2	112719021	0.0875	0.09037	0.09959	0.08038	0.08572	0.02623	0.09463	0.095	0.7841
rs139378193	2	112727229	0.02664	0.02693	0.4291	0.02869	0.02827	1	0.0246	0.02559	0.2208
rs10207237	2	112734855	0.08881	0.09216	0.06855	0.07981	0.08644	0.008924	0.09779	0.09783	1
rs13397194	2	112737979	0.08792	0.09022	0.1535	0.07929	0.08491	0.02134	0.09643	0.09542	1
rs114783679	2	112738584	0.02577	0.02609	0.4069	0.02752	0.02714	1	0.02403	0.02505	0.2115
rs150806833	2	112747986	0.08865	0.09203	0.06786	0.08054	0.08711	0.00979	0.09677	0.09693	0.7918
rs13414207	2	112748053	0.0896	0.09288	0.07095	0.08126	0.08778	0.01073	0.09792	0.09795	1
rs114126070	2	112749293	0.02666	0.02695	0.4293	0.02872	0.02831	1	0.0246	0.02559	0.2208
rs13413103	2	112750695	0.0864	0.09003	0.03807	0.07854	0.08538	0.007174	0.09421	0.09464	0.7822
rs10864895	2	112764394	0.4638	0.4727	0.3136	0.4825	0.4768	0.6634	0.4452	0.4684	0.06045
rs138575760	2	112764470	0.08201	0.1007	5.17E-15	0.07718	0.09274	5.72E-07	0.08681	0.1085	1.92E-09
rs10195619	2	112765562	0.4635	0.4736	0.2474	0.482	0.4775	0.7454	0.4451	0.4694	0.04782
rs13396030	2	112772998	0.07026	0.07089	0.6	0.06488	0.06777	0.1061	0.07564	0.07401	0.7222
rs56361454	2	112774105	0.4497	0.4489	0.935	0.4725	0.4556	0.1722	0.427	0.4417	0.1979
rs7422195	2	112775064	0.4735	0.4682	0.5527	0.4955	0.4737	0.08544	0.4518	0.4622	0.3963
rs71957777	2	112776657	0.06983	0.06987	0.7966	0.0643	0.06723	0.1008	0.07533	0.0725	0.2682
rs13416895	2	112778261	0.07056	0.07057	1	0.06389	0.06687	0.09652	0.07723	0.07425	0.1663
rs112340384	2	112779548	0.0917	0.0881	0.01999	0.0931	0.08998	0.2525	0.0903	0.08622	0.07011
rs72825667	2	112779732	0.07965	0.09837	1.33E-15	0.07641	0.1039	4.63E-14	0.08289	0.09283	0.0005121
rs6735717	2	112780064	0.0704	0.07103	0.6004	0.06443	0.06861	0.03564	0.07636	0.07344	0.272
rs150632843	2	112781454	0.06954	0.07023	0.5958	0.06363	0.06661	0.09544	0.07543	0.07382	0.7221
rs13419523	2	112781917	0.1515	0.1529	0.5512	0.1435	0.1514	0.05714	0.1593	0.1544	0.3128
rs72825671	2	112782371	0.09173	0.08813	0.02008	0.09413	0.0909	0.2531	0.08933	0.08534	0.07025
rs72825672	2	112783327	0.09103	0.0875	0.01981	0.09335	0.09021	0.2525	0.08873	0.08479	0.1146
rs72825673	2	112784082	0.09267	0.08898	0.02075	0.09413	0.0909	0.2531	0.09121	0.08705	0.07048
rs67734060	2	112784544	0.06865	0.06941	0.5911	0.06367	0.0679	0.03298	0.07363	0.07092	0.2617
rs6723394	2	112785696	0.06992	0.0712	0.2986	0.06309	0.06736	0.03079	0.07672	0.07501	0.7245

rs4848958	2	112785775	0.2868	0.4871	6.22E-114	0.3046	0.4911	2.19E-49	0.269	0.4824	8.92E-67
rs2230517	2	112786049	0.06983	0.07049	0.5976	0.06296	0.06599	0.09023	0.07667	0.07496	0.7245
rs13402707	2	112786990	0.07012	0.07076	0.5995	0.06488	0.06901	0.0378	0.07533	0.0725	0.2682
rs6755828	2	112787215	0.1516	0.1531	0.5508	0.1451	0.1527	0.06061	0.1581	0.1534	0.3107
rs10198880	2	112787805	0.1513	0.1523	0.7181	0.1444	0.151	0.1186	0.1582	0.1535	0.311
rs13406390	2	112788095	0.1517	0.1526	0.7195	0.1447	0.1512	0.1196	0.1588	0.154	0.3117
rs13425080	2	112788380	0.152	0.1528	0.7209	0.1457	0.152	0.1227	0.1582	0.1535	0.3114
rs10165940	2	112789092	0.07093	0.07213	0.3099	0.06689	0.06963	0.124	0.07497	0.07463	1

a. Chr=chromosome. b. Location relative to the Human February 2009 (GRC37/hg19) assembly.
c. Obs = observed. d. Exp = expected. Variants highlighted in grey failed both HWE testing and manual quality control.
