# Supplementary material for: Common and Low Frequency Variants in MERTK Are Independently Associated with Multiple Sclerosis Susceptibility with Discordant Association Dependent upon HLA-DRB1*15:01 Status
Source: PLoS Genet. 2016 Mar 18;12(3):e1005853. doi: 10.1371/journal.pgen.1005853 (PMC4798184; doi:10.1371/journal.pgen.1005853)
Supplement: S2 Table — (PDF) [file pgen.1005853.s003.pdf]

Table S2: Apparent size of $TA_nT_n$ repeat expansion in tested samples

| Genotype at rs13414207 | Phenotype | PCR product size 1[a] |
|---|---|---|
| GG | Control | 853 |
| GG | Control | 905 |
| GG | Control | 841 |
| GG | Control | 837 |
| GG | Control | 749 |
| GG | Control | 570 |
| GG | Control | 695 |
| GG | Control | 832 |
| GG | Control | 828 |
| GG | Control | 831 |
| GG | Control | 828 |
| GG | Control | 832 |
| GG | Control | 772 |
| GG | Control | 828 |
| GG | Control | 830 |
| GG | Control | 840 |
| GG | Control | 833 |
| GG | Control | 840 |
| GG | Control | 836 |
| GG | Control | 834 |
| GG | Control | 783 |
| GG | Control | 831 |
| GG | Control | 754 |
| GG | Control | 776 |
| GG | Control | 862 |
| GG | Control | 837 |
| GG | Control | 829 |
| GG | Control | 830 |
| GG | Control | 826 |
| GG | Control | 939 |
| GG | Control | 831 |
| GG | Control | 826 |
| GG | Control | 835 |
| GG | Control | 831 |
| GG | Control | 825 |
| GG | Control | 826 |

| | | |
|---|---|---|
| GG | Control | 935 |
| GG | Control | 838 |
| GG | Control | 836 |
| GG | Control | 830 |
| GG | Control | 830 |
| GG | Control | 838 |
| GG | Control | 827 |
| GG | Control | 829 |
| GG | Control | 828 |
| GG | Control | 829 |
| GG | Control | 830 |
| GG | Control | 820 |
| GG | Control | 819 |
| GG | Control | 836 |
| GG | Control | 835 |
| GG | Control | 835 |
| GG | Control | 833 |
| GG | Control | 878 |
| GG | Control | 763 |
| GG | Control | 763 |
| GG | Control | 828 |
| GG | Control | 825 |
| GG | Control | 844 |
| GG | Control | 837 |
| GG | Control | 835 |
| GG | Control | 835 |
| GG | Control | 842 |
| GG | Control | 838 |
| GG | Control | 835 |
| GG | Control | 833 |
| GG | Control | 830 |
| GG | Control | 887 |
| GG | Control | 841 |
| GG | Control | 748 |
| GG | Control | 840 |
| GG | Control | 839 |
| GG | Control | 831 |
| GG | Control | 887 |
| GG | Control | 841 |
| GG | Control | 748 |
| GG | Control | 840 |
| GG | Control | 839 |

| | | |
|---|---|---|
| GG | Control | 831 |
| GG | Control | 887 |
| GG | Control | 841 |
| GG | Control | 748 |
| GG | Control | 840 |
| GG | Control | 839 |
| GG | Control | 831 |
| GG | Control | 719 |
| GG | Control | 826 |
| GG | Control | 758 |
| GG | Control | 825 |
| GG | Control | 830 |
| GG | Control | 830 |
| GG | Control | 830 |
| GG | Control | 812 |
| GG | Control | 797 |
| GG | Control | 843 |
| GG | Control | 838 |
| GG | Control | 834 |
| GG | Control | 834 |
| GG | Control | 834 |
| GG | Control | 834 |
| GG | Control | 831 |
| GG | Control | 830 |
| GG | Control | 838 |
| GG | Control | 835 |
| GG | Control | 828 |
| GG | Control | 824 |
| GG | Control | 846 |
| GG | Control | 845 |
| GG | Control | 845 |
| GG | Control | 842 |
| GG | Control | 842 |
| GG | Control | 846 |
| AG | Control | 828 |
| AG | Control | 716 |
| AG | Control | 828 |
| AG | Control | 818 |
| AG | Control | 824 |
| AG | Control | 945 |
| AG | Control | 987 |
| AA | Control | 940 |

| | | |
|---|---|---|
| AA | Control | 986 |
| AA | Control | 975 |
| AA | Control | 978 |
| AA | Control | 956 |
| GG | Case | 833 |
| GG | Case | 834 |
| GG | Case | 835 |
| GG | Case | 830 |
| GG | Case | 831 |
| GG | Case | 875 |
| GG | Case | 826 |
| GG | Case | 845 |
| GG | Case | 843 |
| GG | Case | 840 |
| GG | Case | 842 |
| GG | Case | 839 |
| GG | Case | 842 |
| GG | Case | 840 |
| GG | Case | 836 |
| GG | Case | 836 |
| GG | Case | 833 |
| GG | Case | 827 |
| GG | Case | 828 |
| AG | Case | 840 |
| AA | Case | 940 |
| AA | Case | 998 |
| AA | Case | 967 |
| AA | Case | 1,004 |
| AA | Case | 976 |
| AA | Case | 981 |
| AA | Case | 1,007 |
| AA | Case | 977 |
| AA | Case | 1,006 |
| AA | Case | 973 |
| AA | Case | 956 |
| AA | Case | 962 |
| AA | Case | 985 |
| AA | Case | 963 |

a. In general only a single PCR product was detected for any individual
