# Supplementary material for: Common and Low Frequency Variants in MERTK Are Independently Associated with Multiple Sclerosis Susceptibility with Discordant Association Dependent upon HLA-DRB1*15:01 Status
Source: PLoS Genet. 2016 Mar 18;12(3):e1005853. doi: 10.1371/journal.pgen.1005853 (PMC4798184; doi:10.1371/journal.pgen.1005853)
Supplement: S3 Table — (PDF) [file pgen.1005853.s004.pdf]

Table S3: Analysis of linkage between rs13414207 and the intron 4 AluYf4 insertion in two populations

| Population | Genotype at rs13414207 | | |
|---|---|---|---|
| | GG | AG | AA |
| Caucasian Australian | No insertions detected (n=170) | All samples heterozygous for AluY4f insertion (n=14) | Sample homozygous for AluYf4 insertion (n=1) |
| German | No insertions detected (n=161) | All samples heterozygous for AluY4f insertion (n=28) | N/A (no AA samples tested) |
